# Generative and Predictive AI for digital twin systems in manufacturing

**DOI:** 10.3389/frai.2025.1655470

**Published:** 2025-12-17

**Authors:** Dan Dai, Baixiang Zhao, Zhiwen Yu, Pasquale Franciosa, Dariusz Ceglarek

**Affiliations:** 1 School of Computer Science and Digital Technologies, Aston University, Birmingham, United Kingdom; 2 Warwick Manufacturing Group, University of Warwick, Coventry, United Kingdom; 3 China Mobile System Integration Co. Ltd, Beijing, China; 4 School of Computer Science and Engineering, South China University of Technology, Guangzhou, China

**Keywords:** AI-enabled digital twin, Generative AI, Predictive AI, quality assurance, smart manufacturing

## Abstract

The integration of Artificial Intelligence (AI) and Digital Twin (DT) technology is reshaping modern manufacturing by enabling real-time monitoring, predictive maintenance, and intelligent process optimisation. This paper presents the design and partial implementation of an AI-enabled Digital Twin System (AI-DT) for manufacturing, focusing on the deployment of Generative AI (GAI) and Predictive AI (PAI) modules. The GAI component is used to augment training data, perform geometric inspection, and generate 3D virtual testing environments from multiview video input. Meanwhile, PAI leverages sensor data to enable proactive defect detection and predictive quality analysis in welding processes. These integrated capabilities significantly enhance the system's ability to anticipate issues and support decision-making. While the framework also envisions incorporating Explainable AI (EAI), Context-Aware AI (CAI), and Agentic AI (AAI) for future extensions, the current work establishes a robust foundation for scalable, intelligent digital twin systems in smart manufacturing. Our findings contribute toward improving operational efficiency, quality assurance, and early-stage digital-physical convergence.

## 1 Introduction of digital twins

Manufacturing has undergone a significant transformation from manual operations to automated, data-driven systems. Traditional manufacturing relies heavily on static models, rule-based control, and reactive maintenance, which limits adaptability and efficiency. The development of AI-enabled digital twin systems in manufacturing follows a logical progression from rigorous design verification and validation models, which include both the integration and co-evolution of products and processes (Tolio et al., 2010). These models are further extended to consider the product lifecycle (Maropoulos, 2010). Then, progressing to advanced sensing is necessary for effective process monitoring with capabilities to enable root cause analysis of quality defects (Ding et al., 2003). This ultimately leads to AI-driven control and autonomy of manufacturing processes (Ding et al., 2019; Gao et al., 2024). Early work on systematic validation frameworks established the conceptual foundation for virtual models of processes and products (Maropoulos, 2010; Tolio et al., 2010), while research in sensing led to (i) development of novel sensors for in-line/in-process monitoring of manufacturing systems (Shi, 2006); (ii) determining placement of in-line monitoring station(s) within a multi-stage manufacturing systems which is then synergistically integrated with measurement coverage analysis for more effective process monitoring, for e.g., reducing mean-time-to-detection by advancing process monitoring from off-line inspection toward in-line and in-process monitoring (Modoni et al., 2022) and system diagnosability (Ding et al., 2003); and (iii) increase monitoring data: variety (for e.g., ranging from categorical to spatio-temporal data) and volume (from small samples to 100% inspection rate) which are collected in-process with sufficient fidelity (i.e., within the required accuracy, repeatability and reproducibility) and veracity (i.e., accurate reflections of the process “defect condition” patterns). These advances provided the real-time input needed for both high fidelity and veracity analysis of the manufacturing process (Babu et al., 2019; Wang et al., 2021).

Subsequent methodologies for root cause analysis (RCA) and process variation modeling have provided a robust foundation for diagnosing product quality issues and quantifying sources of process variability, and as such evolved into critical components of intelligent manufacturing systems (Ceglarek et al., 2004; e Oliveira et al., 2023). RCA methodologies facilitate the systematic identification of latent defect drivers, while variation modeling quantifies stochastic process behavior and parameter sensitivities (Ceglarek et al., 1994; Franciosa et al., 2020). These analytical frameworks, when synergistically integrated with deep learning architectures and statistical reasoning techniques for predictive quality estimation (Sinha et al., 2020) and closed-loop process control, enable the development of high-fidelity digital twins (Soori et al., 2023). Simultaneously, the emergence of smart manufacturing theme supported by “Factory of the Future” and then Industry 4.0/5.0 and the “twin transition” (i.e., integration of digital and green transformations) initiatives (European Commission, 2020; Thelen et al., 2023) has further reinforce the need for real-time process monitoring, predictive analytics, and intelligent decision-making, i.e., enablers that currently drive the development of Digital Twin (DT) Technology (Wang et al., 2021; Villalonga et al., 2021).

Digital twin (DT) systems have emerged as a groundbreaking innovative framework in manufacturing (Leng et al., 2021; Soori et al., 2023), offering a virtual replica of physical processes and systems to enable real-time process monitoring (Franciosa et al., 2020; Ceglarek et al., 2015), optimization (Thelen et al., 2023), process control (Stavropoulos et al., 2021; Stavropoulos and Sabatakakis, 2024), and decision-making (Bocklisch Franziska, 2023). By integrating advanced sensor networks (Javaid et al., 2021), robust data management (Schadt et al., 2010), and artificial intelligence (AI)-driven analytics (Ertel, 2024), digital twins empower manufacturers to simulate, predict, and proactively resolve potential issues. While significant progress has been made in enabling in-line and in-process monitoring, comparatively fewer studies have addressed control-oriented digital twins, and even fewer have systematically examined the robustness of their underlying models when faced with uncertainties such as sensor noise, parameter variability, and environmental disturbances (Stavropoulos et al., 2021). Addressing these challenges remains critical for ensuring reliable and resilient digital twin deployment in industrial environments.

AI-driven approaches are expected to revolutionize digital twin technology by significantly expanding current reliance on traditional simulations by enhancing real-time decision-making, predictive modeling, and process optimization. Unlike traditional simulation-based methods (Mourtzis et al., 2015), which often rely on predefined scenarios and assumptions that limit responsiveness due to time-consuming calculations and dynamic environments (Tao et al., 2024), AI has the potential to learn from vast amounts of historical information, physical simulation and real-time monitoring data to generate accurate predictions, adapt dynamically to changing conditions, and provide context-based insights and ultimately better informed decisions (Nti et al., 2022). AI-driven defect detection (Ren et al., 2022), predictive maintenance (Achouch et al., 2022), and quality assurance (Sinha et al., 2020) can leverage various sources of data, information, and knowledge without relying only on a single resource-intensive simulation, offering manufacturers a more scalable and efficient approach.

In the context of current manufacturing trends (i.e., Industry 4.0 and twin transition) where manufacturing ecosystems are increasingly characterized by cyber-physical integration, interoperability, and data-driven decision-making (Ceglarek, 2014; Herrera Vidal, 2021), the limitations of traditional simulation-based digital twins in addressing scalability, responsiveness, and contextual awareness have become evident. To address these challenges, we propose an AI-enabled Digital Twin (AI-DT) framework that operationalizes key enablers of Industry 4.0/twin transition through the integration of advanced AI modalities: Generative AI for synthetic data augmentation and design-space exploration; Predictive AI for real-time forecasting and anomaly detection; Explainable AI for model interpretability and traceability; Context-Aware AI for environment-sensitive adaptability; and Autonomous Agentic AI for decentralized, self-governing control and decision-making. This composite AI-DT architecture enhances the semantic interoperability, cognitive autonomy, and adaptive intelligence of digital twins, thereby supporting resilient, self-optimizing manufacturing in line with Industry 4.0 and “twin transition” objectives.

## 2 AI-enabled digital twin framework

**Figure 1:**
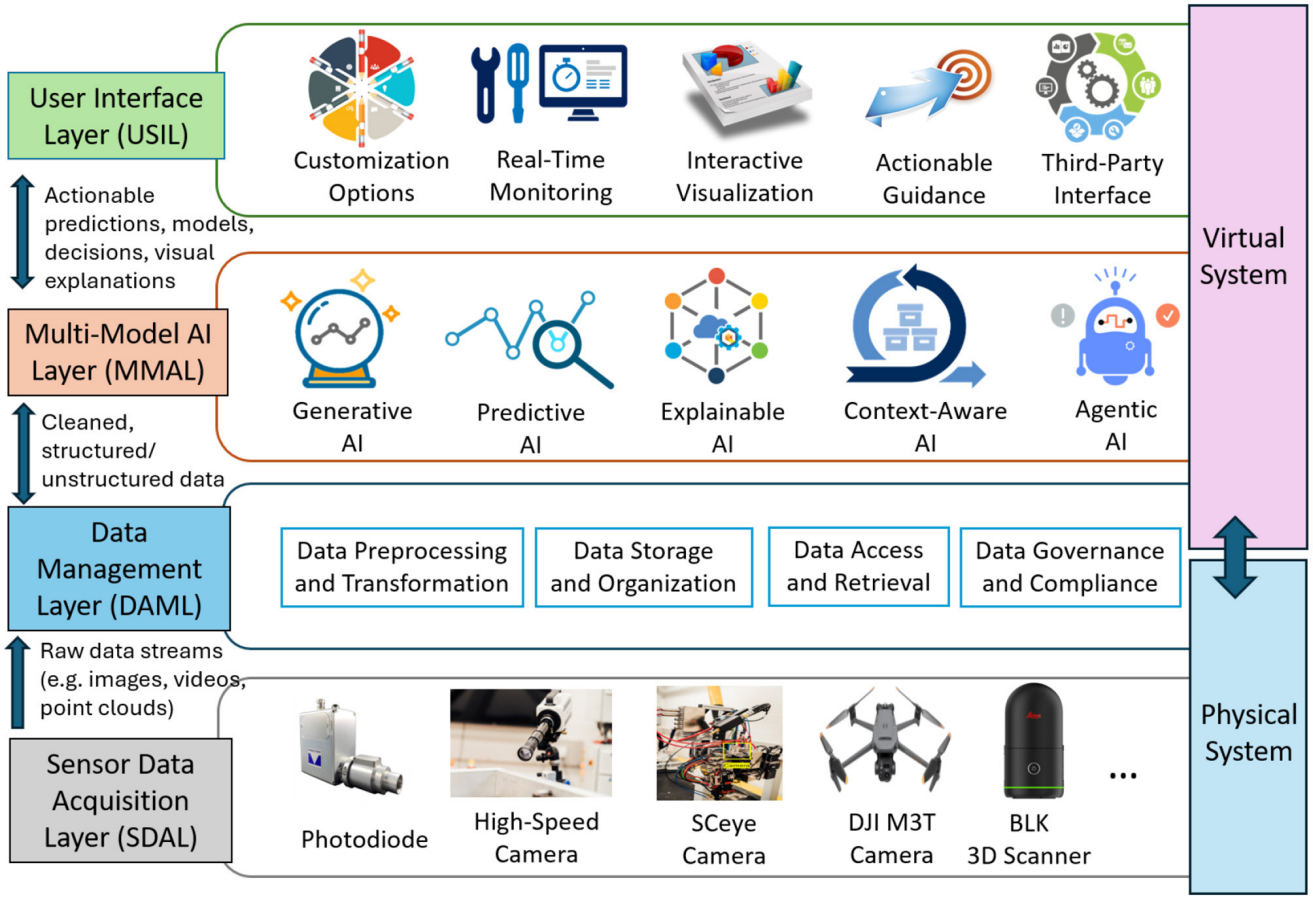
Framework of the AI-enabled Digital Twin (AI-DT) System for Manufacturing. The system is composed of four core layers: the Sensor Data Acquisition Layer collects multimodal inputs (e.g., photodiodes, high-speed cameras, 3D scanners); the Data Management Layer handles data preprocessing, storage, access, and compliance; the Multi-Model AI Layer integrates diverse AI modules, the User Interface Layer provides visualization, monitoring, and third-party interfacing capabilities. This modular and scalable architecture bridges physical and virtual systems to enable intelligent, real-time manufacturing insights.

The framework provided in Figure 1 outlines a comprehensive and modular architecture for the AI-enabled Digital Twin (AI-DT) system. It integrates physical and virtual systems, ensuring seamless interaction between real-world processes and their digital counterparts. The framework is designed to leverage cutting-edge technologies like sensors, advanced data management, AI and interactive user interfaces.

**1. Sensor data acquisition layer (physical system, SDAL)** forms the foundation of a digital twin system by accurately mirroring the physical system through real-time acquisition of diverse types of data from various sensors. This layer plays a critical role in monitoring, analyzing and simulating real-world processes by providing high-fidelity, precise, and contextually relevant inputs. The SDAL primarily focuses on data collection and transmission, serving as the entry point for real-world data into the virtual ecosystem. While the sensor hardware (e.g., photodiodes, cameras, and 3D scanners, et al.) is typically pre-selected and fixed based on system requirements, the emphasis lies on how data is efficiently captured and transmitted to meet system demands. This ensures the quality and timeliness of the data flow that underpins the accuracy and effectiveness of the Digital Twin.

**2. Data management layer (bridging physical and virtual systems, DAML)** acts as the bridge between the physical and virtual domains, responsible for processing, organizing, and managing the data collected by the sensors. Its main objective is to handle the vast amounts of data generated by the SDAL and make it usable for the Multi-Model AI Layer and the User Interface Layer.

**3. Multi-model AI layer (virtual system, MMAL)** is the core intelligence engine of the AI-DT system. It leverages multiple AI techniques to process different kinds of data, generate insights, predict outcomes, and provide actionable recommendations. This layer operates on data provided by the DAML, supports real-time and proactive decision-making in manufacturing.

**Generative AI (GAI)** helps simulate scenarios, generate synthetic data, create virtual representations of real-world systems, and is often the starting point in creating a Digital Twin system, it lays the foundation for building the virtual model of the physical system (Gozalo-Brizuela and Garrido-Merchan, 2023; Bandi et al., 2023; Feuerriegel et al., 2024). The main tasks include: (1) Synthetic Data Generation (Lu et al., 2023): it generates realistic training datasets to augment defect detection models, addressing challenges like data scarcity or class imbalances. (2) 3D Model Reconstruction (Liu et al., 2024): utilizes video or sensor data to generate high-resolution 3D models of manufacturing components for inspection and 3D environment building. GAI can effectively simulate manufacturing scenarios to test and validate predictive models without disrupting real-world operations.

**Predictive AI (PAI)** focuses on forecasting future system states, such as defects, based on historical and real-time sensor data. Its primary role is to analyse and infer probable outcomes, supporting proactive decision-making (e.g., predicting porosity or spatter formation based on welding video). The key tasks include (1) Defect Classification (Dai et al., 2024): categorizes defects into specific types (e.g., spatter, porosity, cracks) based on labeled data and predefined classes. This involves analyzing sensor data and assigning defect categories to improve the efficiency of quality inspection. (2) Defect Detection (Ren et al., 2022): identifies the presence and location of defects in manufacturing components using sensor data, video streams, or 3D models. It employs computer vision and sensor fusion techniques to locate defect areas in real-time. (3) Defect Prediction (Liu et al., 2022): predicts the occurrence and type of defects based on process parameters, sensor data, and historical defect records.

Beyond defect classification, detection, and prediction, PAI methods are also widely applied to non-intrusive or indirect feature measurement. For example, in welding, PAI can infer weld quality metrics without destructive testing, while in additive manufacturing, it can estimate over-deposition or surface geometry deviations from sensor data (Stavropoulos and Sabatakakis, 2024; Bourlesas et al., 2024). This extended role demonstrates the flexibility of PAI in providing quality assurance through indirect, real-time measurements that avoid part removal or offline inspection.

**Explainable AI (EAI)** is critical for interpreting predictions and decisions made by the system, fostering trust and transparency, it provides insights into the “why” and “how” behind system behaviors, which is important for audits, compliance, and user trust (Hrnjica and Softic, 2020; Chen, 2023; Ahmed et al., 2022). The key tasks include (1) Model Explainability (Dai et al., 2024; Li et al., 2018): it could provide clear explanations of how AI models arrive at their classifications or predictions. (2) Causal Analysis (e Oliveira et al., 2023): Identifies the underlying factors influencing AI decisions by establishing cause-and-effect relationships between input variables and predictions. This helps in understanding why specific defects occur, how process variations impact outcomes, and what corrective actions can mitigate identified issues. (3) Trust Building (Choung et al., 2023): It increases trust in AI systems by offering insights into decision-making processes, especially in high-stakes scenarios.

**Context-aware AI (CAI)** ensures adaptability and robustness in real-time decision-making, making predictions more relevant and actionable by incorporating environmental and operational variations (Hribernik et al., 2021). The key tasks include: (1) Dynamic Decision-Making (Villalonga et al., 2021): adjusts recommendations based on current process parameters (e.g., speed, material type, temperature) and evolving conditions. (2) Context Integration (Reiman et al., 2021): incorporates external factors such as environmental conditions, operator inputs, or production goals to tailor AI actions. (3) Real-Time Feedback (Pantelidakis et al., 2022): provides adaptive feedback loops for optimizing processes as they occur.

**Agentic AI (AAI)** represents an autonomous decision-making agent that can act based on insights from modules such as GAI, PAI, EAI, and CAI, shown in Table 1. AAI is designed to close the loop by executing decisions. It empowers the system to act without human intervention, enabling full autonomy in complex environments (Acharya et al., 2025). The key tasks include: (1) Autonomous Operations (Leng et al., 2023): executes routine tasks like defect classification, report generation, or process adjustments without human input. (2) Decision Support (Psarommatis and Kiritsis, 2022): provides ranked recommendations or options for operators to choose from, balancing automation with human oversight. (3) Learning and Adaptation (Li C. et al., 2022): continuously learns from feedback and evolving scenarios to improve its decision-making capabilities. For example, an Agentic AI module in a welding application can operate as a self-correcting welding robot that autonomously adjusts process parameters, such as laser power, welding speed, or focal position, in response to defect signals. Unlike traditional automation systems that follow predefined rules, this self-correcting behavior allows the robot to learn from sensor feedback and adapt dynamically to process variations, ensuring consistent weld quality without human intervention.

**Table 1:** Summary of five core AI types integrated within digital twin systems.

**AI type**	**Definition**	**Key applications**	**Relevance**	**Challenges**
Generative AI (GAI)	Generates synthetic data, simulates scenarios, and creates virtual representations of real-world systems.	Synthetic data generation for training AI models, 3D model generation, and process simulation.	Enhances training data and scenario simulation in data-scarce environments.	Generating realistic and domain-specific synthetic data.
Predictive AI (PAI)	Analyzes data to forecast future states, identify trends, and predict potential issues.	Predictive maintenance, defect detection, and system performance forecasting.	Provides proactive insights that enable operators or autonomous agents to anticipate and prevent failures.	Accurately predicting complex, non-linear system behaviors.
Explainable AI (EAI)	Provides interpretability and transparency to AI models, explaining decisions and outputs.	Root cause analysis, auditing AI decisions, and regulatory compliance.	Builds trust in the system by making AI outputs interpretable.	Balancing model interpretability with prediction accuracy.
Context-Aware AI (CAI)	Accounts for environmental and operational contexts to make adaptive decisions.	Adaptive process control, real-time parameter optimization, and environmental adjustments.	Ensures consistent system performance under varying conditions.	Identifying and incorporating relevant contextual factors.
Agentic AI (AAI)	Executes autonomous decision-making and control actions based on insights from GAI, PAI, EAI, and CAI.	Autonomous defect response, real-time process control, corrective decision execution.	Enables Digital Twin systems to self-adjust, optimize, and respond autonomously without human intervention.	Ensuring safe and reliable autonomy under complex conditions.

Each AI type is characterized by its functional definition, key applications, strategic relevance, and challenges.

**4. USer interface layer (virtual system, USIL)** forming the virtual interface of the AI-DT System, acts as the primary point of interaction between operators and the decisions (Banaeian Far and Imani Rad, 2022). This layer is designed to ensure seamless communication, real-time feedback, and actionable insights for effective decision-making and process optimization. In addition to its role as the primary interaction layer, the USIL enables bi-directional engagement with both MMAL. For example, operators can initiate tasks such as 3D reconstruction and synthetic data augmentation through the USIL, directly visualizing outputs generated by GAI. Likewise, such as defect classification, detection outputs, and predictive trends (e.g., spatter evolution) from PAI's results, are presented via real-time dashboards, supporting informed decision-making. Operator feedback provided through the USIL can be fed back into GAI and PAI, like by refining simulation parameters, adjusting predictive thresholds, or prioritizing specific defect categories. This continuous feedback loop ensures that AI outputs are not only accessible and interpretable but can also be iteratively improved and adapted within production workflows.

## 3 Designing and building test system

In this paper, we use welding as a case study, present a comprehensive framework for designing and implementing a DT system, our approach integrates data collection, advanced AI to develop and apply for industrial processes. The primary objective of this virtual system is to enable operators to carry out their tasks within an augmented or virtual reality environment. Furthermore, evaluate whether welding products meet the required tolerances based on specified process parameters. This framework not only bridges the gap between theory and practical application but also demonstrates the potential of DT systems to transform manufacturing processes by improving operational efficiency and product quality.

**Figure 2:**
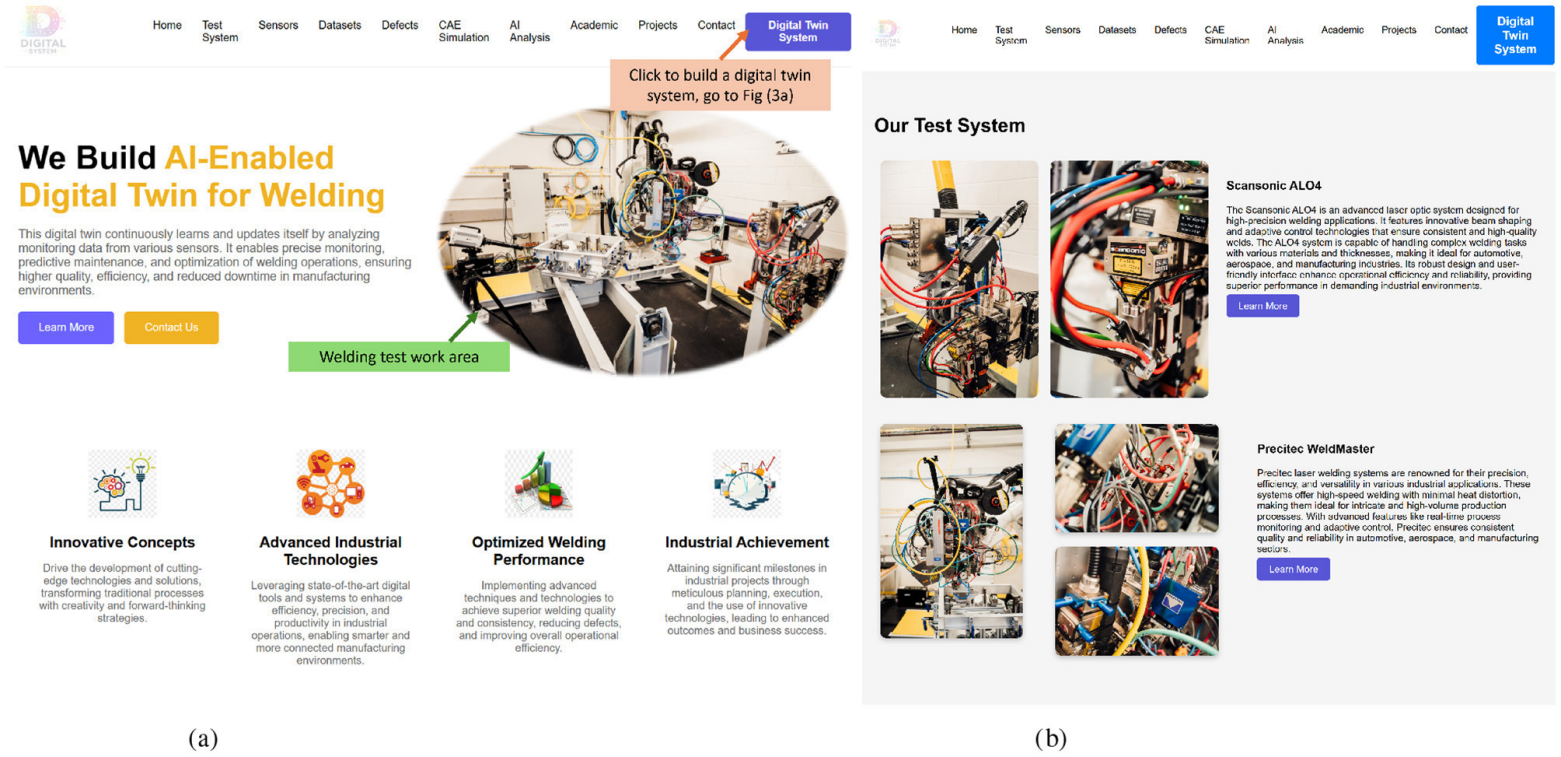
**(a)** A web-based interface showcasing the integration of AI modules, sensor data, and digital twin functionalities for welding process optimisation and defect monitoring. **(b)** The physical welding system setup comprising the Scansonic ALO4 and Precitec WeldMaster modules used for laser welding experiments and data acquisition to support digital twin development and evaluation. **(a)** AI-enabled digital twin system for welding. **(b)** Welding test system for AI-DT.

**Figure 3:**
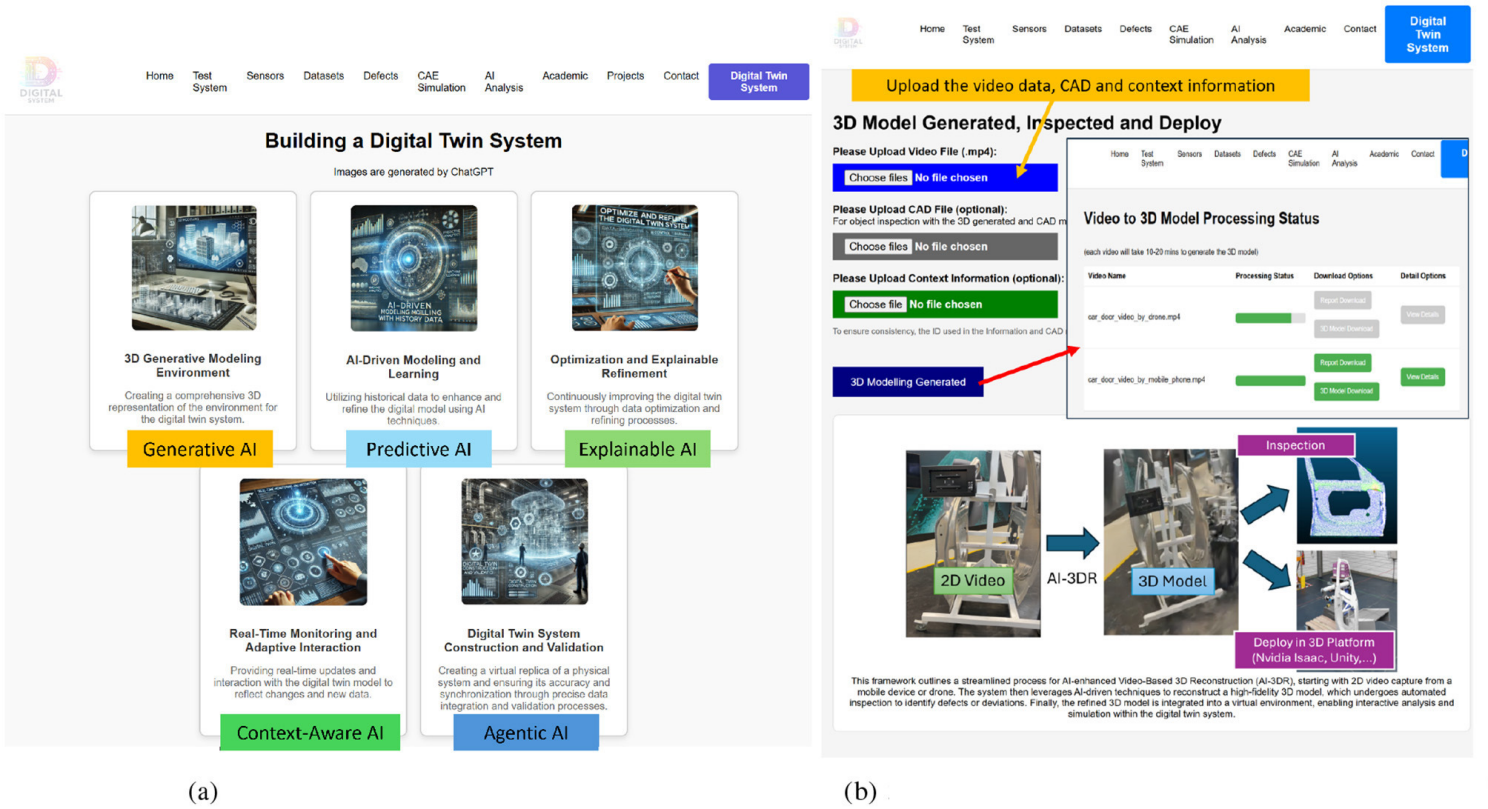
**(a)** Modular AI Interface of the Digital Twin System, showcasing the integration of Generative AI, Predictive AI, Explainable AI, Context-Aware AI, and Agentic AI to support end-to-end quality assurance and autonomous decision-making. **(b)** Interactive 3D Model Generation Interface, illustrating the workflow for uploading video data (e.g., from mobile phones or drones), converting it into high-fidelity 3D models, and deploying it within the Digital Twin environment for inspection and validation. **(a)** AI-driven digital twin system modules. **(b)** 3D models generated interface powered by the GAI module.

Figure 2a serves as the home page of the AI-DT System for welding, offering an overview of the welding system (Figure 2b), sensors, datasets, defects and AI analysis. Building on this foundational overview, the detailed technical framework (Figure 3a) provides a structured methodology for the design and implementation of the AI-DT system. This framework leverages AI-driven methodologies to enhance modeling, monitoring, and decision-making capabilities, ensuring a seamless interaction between physical and virtual systems. (1) 3D Generative Modeling Environment starts with the creation of a 3D virtual model that accurately replicates the welding environment. Using Generative AI, the model is constructed from multi-view videos, integrating spatial, geometric, and contextual data for an immersive digital representation. (2) AI-Driven Modeling and Learning is predicted using Predictive AI while incorporating physics-informed constraints to ensure realistic process simulations. AI learns from historical welding data, simulation outputs, and real-world observations, enabling precise quality assessment and defect forecasting. (3) Optimization and Explainable Refinement undergoes iterative root cause analysis and performance optimization through Explainable AI. This step ensures that AI decisions, such as defect predictions and recommended process adjustments, are transparent and interpretable, fostering trust in automated decision-making while improving accuracy over time. (4) Real-Time Monitoring and Adaptive Interaction remains dynamic through real-time monitoring, capturing live data from sensors, video feeds, and contextual information. Context-Aware AI adapts the digital twin's behavior in response to changes in environmental conditions, process variations, or external influences, ensuring continuous feedback and actionable insights. (5) Digital Twin System Construction and Validation integrates Agentic AI for autonomous decision-making, allowing it to proactively adjust parameters, detect defects, and implement corrective actions without direct human intervention. This ensures that the digital twin can self-optimize and maintain peak efficiency in dynamic operational environments.

### 3.1 3D generative modeling environment for welding object reconstruction

To create a virtual environment that accurately replicates the physical one, it is essential to incorporate detailed 3D models of various objects, including their geometric shapes, functional attributes, and precise spatial locations. This process captures the structural and operational characteristics of each object, ensuring the virtual representation aligns seamlessly with the physical environment. Such fidelity is critical for enabling effective interaction, analysis, and simulation within the virtual space. There are multiple methods to generate 3D models, including: (1) Computer-Aided Design (CAD): CAD models are manually created by professionals based on design specifications, blueprints, or technical drawings. (2) Reverse Engineering: This approach involves reconstructing a digital 3D model from a physical object through the use of technologies such as 3D scanning and mesh processing. It is commonly applied to replicate existing parts, perform quality analysis, or generate digital representations of legacy components without existing CAD data (Helle and Lemu, 2021). (3) Artificial Intelligence Generated Content: AI techniques such as Image-to-3D reconstruction [e.g., Neural Radiance Fields (Mildenhall et al., 2021)], Text-to-3D [e.g., DreamFusion (Poole et al., 2022)], 3D Gaussian Splatting (3DGS) (Kerbl et al., 2023), and Direct3D (Wu et al., 2024) allow automated generation of 3D models from inputs like images, sketches, or point clouds. Among these, GAI is particularly promising for its ability to handle diverse input formats and automate the 3D model generation process. As AI technologies evolve, GAI-generated 3D models are expected to become a primary approach due to their scalability, efficiency, and adaptability.

**Figure 4:**
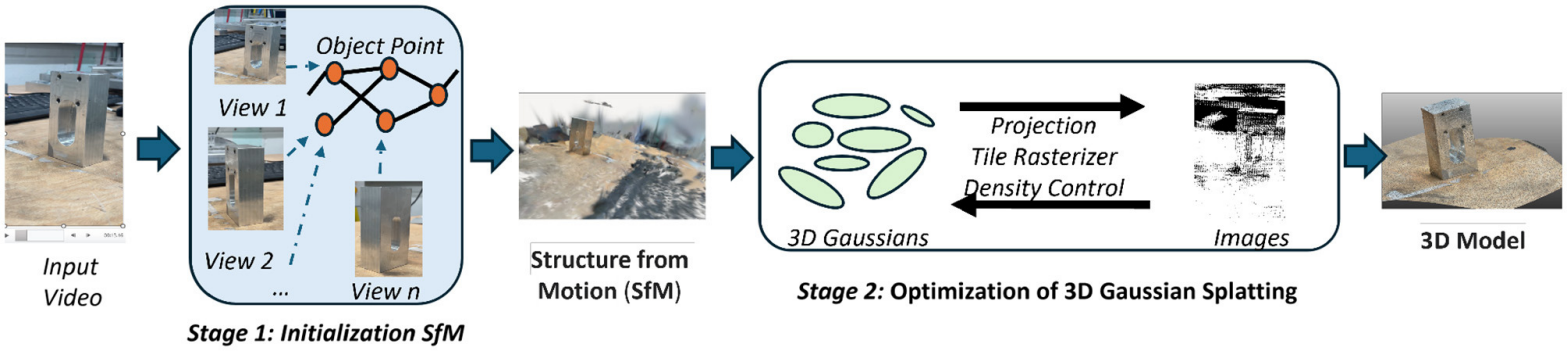
Framework of the AI-enhanced Video-to-3D Reconstruction (AI-3DR) Model for GAI modular. The pipeline includes Stage 1: Initialization using Structure from Motion (SfM), where multiple video views are used to extract object points and build a sparse 3D structure, followed by Stage 2: Optimization via 3D Gaussian Splatting, which refines the geometry through projection, tile rasterisation, and density control. The final output is a high-fidelity 3D model suitable for virtual inspection, simulation, and digital twin integration.

**AI-enhanced video-to-3D reconstruction (AI-3DR):** Our system focuses on leveraging video data to generate 3D models for inspection and deployment into a virtual environment, the system page in Figure 3b. The AI-enhanced Video-to-3D Reconstruction process includes: (1) Video Acquisition: Capturing video data from various perspectives using mobile cameras or drones. (2) 3D Model Generation: The AI-3DR module converts input video from mobile devices, drones, or fixed cameras into accurate and high-fidelity 3D models. As illustrated in Figure 4, the reconstruction process involves two main stages. Stage 1 performs Structure from Motion (SfM), which extracts object points by analyzing multiple video frames from different viewpoints (e.g., View 1, View 2, and View n), resulting in a sparse 3D structure that captures the global geometry of the object. Stage 2 involves the optimisation of this structure using 3D Gaussian Splatting (3DGS) (Kerbl et al., 2023), which enhances spatial fidelity through a series of steps including projection, tile rasterisation, and density control. These steps refine the point cloud and produce a continuous surface representation, ultimately generating a dense, photorealistic 3D model suitable for downstream tasks such as defect inspection, simulation, or integration into digital twin environments. This workflow highlights the integration of motion capture and advanced AI-driven optimization for precise 3D reconstruction. (3) Inspection and Deployment: By analyzing the generated 3D models for defects or deviations, our system will generate a quality analysis report to show the local area that contains a large degree of deformation.

**Quantitative evaluation of 3D reconstruction:** To evaluate the fidelity of the AI-3DR generated 3D models, we perform a Cloud-to-Cloud (C2C) distance analysis between the AI-3DR output point cloud $P=\left\{p_{1},p_{2},\ldots ,p_{N}\right\}\subset {\mathbb{R}}^{3}$ and a ground-truth CAD model point cloud $Q=\left\{q_{1},q_{2},\ldots ,q_{M}\right\}\subset {\mathbb{R}}^{3}$. For each point *p*_*i*_∈*P*, the shortest Euclidean distance to the reference point cloud *Q* is computed as:


$$\begin{array}{l} d\left(p_{i},Q\right)=\underset{q_{j}\in Q}{\min}||p_{i}-q_{j}|{|}_{2} \end{array}$$ (1)

The set of all such distances defines the C2C deviation:


$$\begin{array}{l} D_{C2C}=\left\{d\left(p_{i},Q\right)|p_{i}\in P\right\} \end{array}$$ (2)

From this, we compute the Mean distance, Standard deviation, and Maximum observed distance:


$$\begin{array}{l} \mu_{C2C}=\frac{1}{N}\overset{N}{\underset{i=1}{\sum }}d\left(p_{i},Q\right) \end{array}$$ (3)

$$\begin{array}{l} \sigma_{C2C}=\sqrt{\frac{1}{N}\overset{N}{\underset{i=1}{\sum }}{\left(d\left(p_{i},Q\right)-\mu_{C2C}\right)}^{2}} \end{array}$$ (4)

$$\begin{array}{l} d_{\max}=\underset{i}{\max}d\left(p_{i},Q\right) \end{array}$$ (5)

To contextualize the geometric accuracy, we compare the mean C2C distance to the object's bounding box diagonal as Relative Error (RE):


$$\begin{array}{l} \text{Diagonal}=\sqrt{L^{2}+W^{2}+H^{2}} \end{array}$$ (6)

$$\begin{array}{l} \text{Relative Error}=\frac{\mu_{C2C}}{\text{Diagonal}}\times 100\% \end{array}$$ (7)

where *L, W, H* are the length (height), width, and thickness/depth of the object, the resulting Relative Error is a percentage expressing the average deviation with respect to object size, this low relative error indicates high geometric fidelity, suitable for quality inspection and Digital Twin deployment in industrial settings.

For our car door, the dimensions are *width* = 1, 330*mm, height* = 1, 470*mm, thickness* = 280*mm*. The results from our evaluation, based on C2C analysis shown in Table 2. The mean deviation of 22.32 mm and standard deviation of 14.70 mm indicate a relatively tight spread in deviation, though the tail (max 105 mm) suggests some outlier points. The dominant deviation range (10–30 mm) indicates that the majority of points in the AI-3DR model deviate from the ground-truth CAD model by only 10 to 30 mm, reflecting consistent and accurate reconstruction across most of the object's surface. A relative error of 1.11% with respect to object scale demonstrates that the reconstructed geometry is highly accurate, validating its use in precision manufacturing workflows such as inspection, virtual assembly, and process optimisation.

**Table 2:** Quantitative results of C2C analysis between AI-3DR reconstruction and CAD model.

**Metric**	**Symbol**	**Value**
Mean C2C distance	μ_*C*2*C*_	22.32 mm
Standard deviation	σ_*C*2*C*_	14.70 mm
Maximum distance (approx.)	*d*_max_	105 mm
Dominant deviation range	—	10–30 mm
Relative error	RE	1.11%

**Figure 5:**
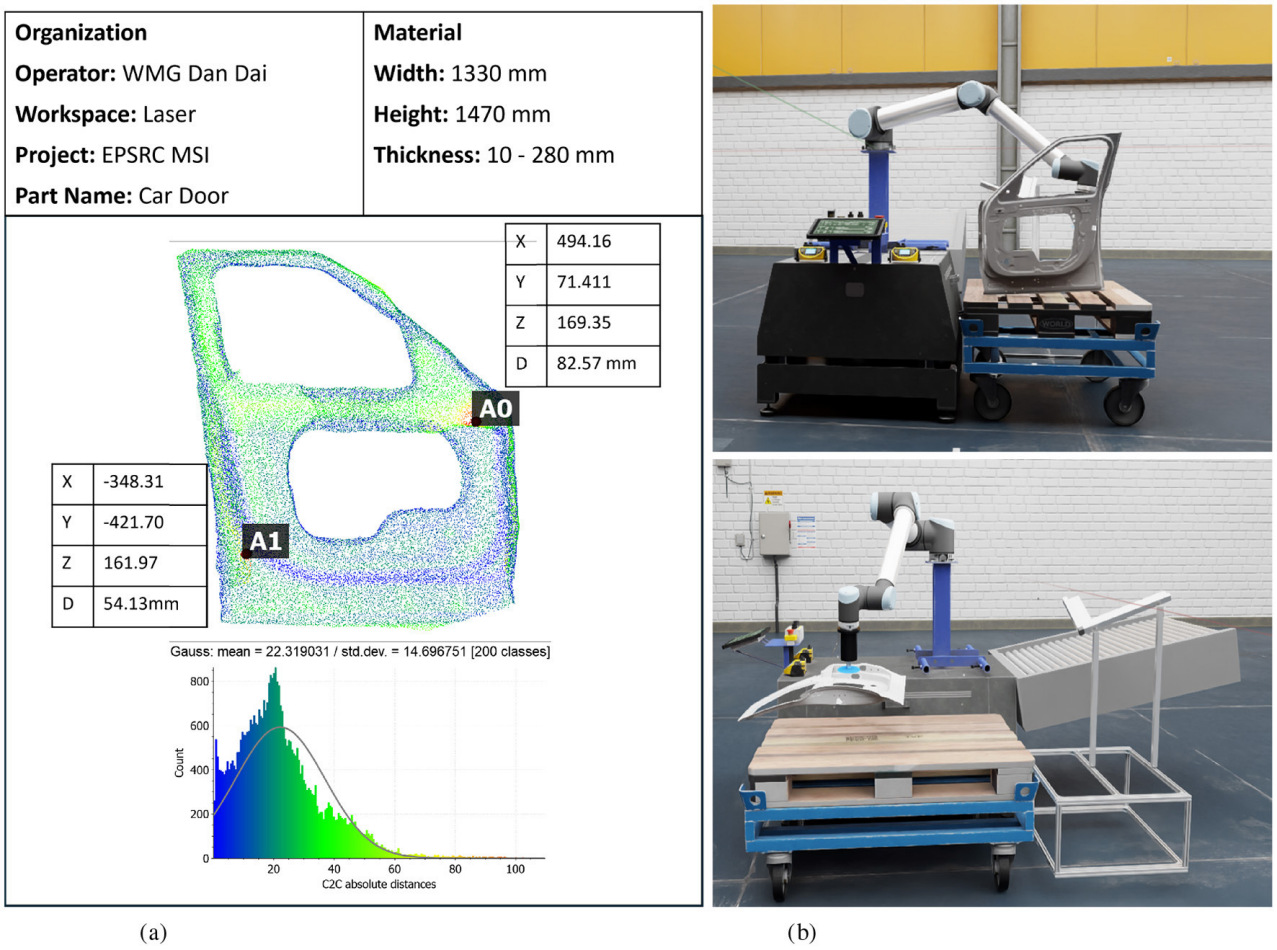
**(a)** Quality analysis report of a car door based on the AI-3DR-generated 3D model for GAI modular. The report includes detailed spatial deviation measurements (e.g., at points A0 and A1), part dimensions, and a histogram of cloud-to-cloud (C2C) distance deviations, supporting defect detection and dimensional validation. **(b)** Virtual integration of the 3D Car Door Model into a digital twin platform, enabling robotic simulation, in-process inspection, and quality validation in a smart manufacturing environment. **(a)** Quality analysis report about the car door. **(b)** Virtual integration of 3D car door model.

To provide practical insights into the system's deployment readiness, we evaluated the processing times for key modules. The video-to-3D reconstruction pipeline, which includes frame extraction, feature matching, point cloud generation, and mesh refinement, takes approximately 12–15 min per 10-second video, depending on resolution and scene complexity. The Quality Analysis Report (Figure 5a) presents a point cloud-based inspection of a car door, detailing dimensional measurements, deviations, and statistical analysis. The report includes key material specifications such as width, height, and thickness, along with precise spatial coordinates (X, Y, Z) and deviation (D) values at critical points (A0, A1). The bottom histogram represents the absolute distance distribution C2C, illustrating the deviation pattern across the scanned model. This analysis enables defect detection, structural validation, and process optimization in laser-based manufacturing environments. The 3D car door model can be imported into a virtual platform (Figure 5b), allowing simulation, inspection, and process validation within a digital twin environment. The system utilizes AI-driven 3D reconstruction to replicate the physical car door in a realistic virtual workspace, allowing for robotic path planning, quality analysis, and automated assembly simulations. This integration enhances manufacturing precision, defect analysis, and workflow optimization in smart factory settings.

### 3.2 AI-driven modeling and learning for welding defect detection

**Figure 6:**
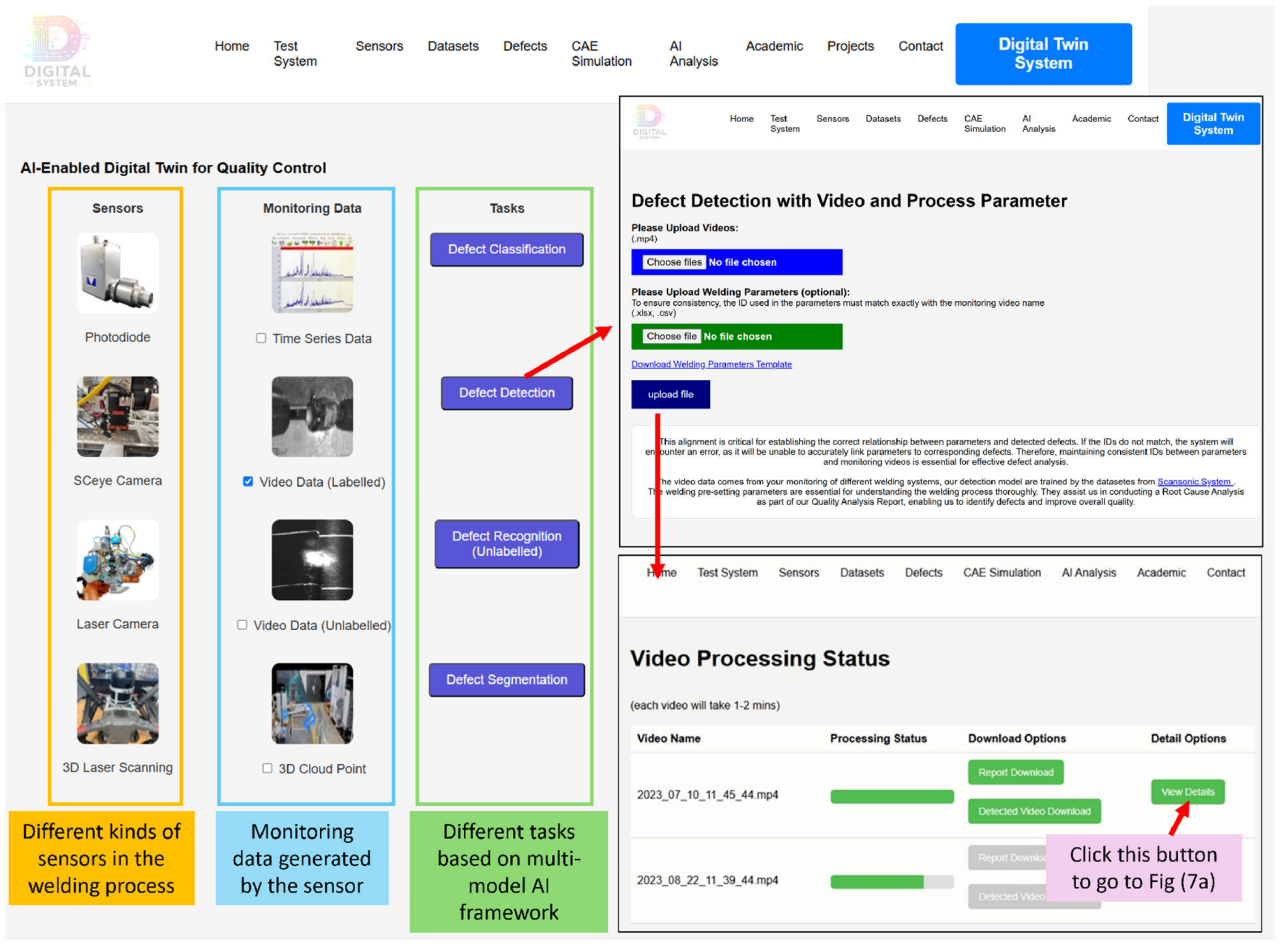
AI-driven defect identification workflow for Welding Quality Control and PAI modular. The interface guides users through uploading sensor data and process parameters, initiating defect detection, and visualizing results. The processing status and outputs—such as detection videos and downloadable defect reports—enhance traceability and support actionable insights for quality assurance.

AI-driven modeling and learning form the backbone of a robust digital twin system by combining advanced machine learning techniques and physics-informed constraints to ensure high accuracy and physical consistency. The system integrates multi-modal sensor data, including photodiodes, high-speed cameras, laser scanners, and 3D laser scanning, to capture monitoring data such as time series, video, and 3D point clouds. Users upload their data and process parameters, which are processed by AI-based defect classification, detection, and segmentation models (Figure 6). We will demonstrate this AI-driven modeling and learning through defect detection.

**Semi-supervised welding defect detection (SSWD):** The process of annotating welding defect data is both labor-intensive and time-consuming, especially in industrial environments where large volumes of unlabeled data are continuously generated. To address this challenge, we propose a semi-supervised defect detection framework based on a Transformer-based teacher-student architecture (Hu et al., 2023), designed to leverage labeled and unlabeled data effectively. The framework comprises the Detection Transformer Teacher and the Detection Transformer Student two core components. The teacher model is trained using a limited set of labeled welding data, the student model is trained primarily on the unlabeled data, using the teacher's predictions as soft targets. Through this teacher–student paradigm, the system effectively propagates reliable knowledge from labeled to unlabeled domains, improving defect detection accuracy while significantly reducing the dependency on manual annotation. The integration of transformer architectures ensures that both spatial and contextual information is preserved, further enhancing the system's generalization capabilities in real-world welding scenarios.

**Quantitative evaluation of defect detection:** To assess the performance of defect detection models on labeled data, we adopt the Average Precision at IoU = 0.50 (AP_50_) metric. AP_50_ measures the model's accuracy in detecting and localizing defects by evaluating how well the predicted bounding boxes align with ground-truth annotations, based on a fixed Intersection over Union (IoU) threshold of 0.50. Mathematically, AP is computed as the area under the precision–recall curve:


$$\begin{array}{l} {\text{AP}}_{50}=\overset{1}{\underset{0}{\int }}P\left(R\right)dR \end{array}$$ (8)

where *P*(*R*) denotes the precision as a function of recall *R*. In practice, this integral is approximated by a summation over all discrete recall levels at which precision drops occur. A higher AP_50_ value reflects better performance, indicating that the model maintains high precision on a wide range of recall levels, a crucial property for reliable defect identification in manufacturing systems.

In our semi-supervised welding defect detection framework, our dataset consists of 450 welding videos, which collectively yield approximately 146,000 image frames, of which only 1,247 are manually labeled. we also compare with Soft Teacher (Xu et al., 2021), Dense Teacher (Zhou et al., 2022), PseCo (Li G. et al., 2022), and ARSL (Liu et al., 2023), these models are trained using 1,247 labeled images and 145,138 unlabeled images. Table 3 presents a comparative evaluation of AP_50_ across those five semi-supervised defect detection methods. Despite the limited labeled data, our SSWD achieves a strong performance, with an AP_50_ of 85.54%. This result highlights the effectiveness of our Teacher–Student architecture in leveraging large-scale unlabeled data to improve detection accuracy, while significantly reducing reliance on manual annotation.

**Table 3:** Comparison of AP_50_ performance across semi-supervised defect detection methods.

**Method**	**AP_50_ (%)**	**Method**	**AP_50_ (%)**
Soft teacher (Xu et al., 2021)	82.03	Dense teacher (Zhou et al., 2022)	82.61
PseCo (Li G. et al., 2022)	81.67	ARSL (Liu et al., 2023)	83.94
**SSWD (ours)**	**85.54**

Bold values indicate the best AP_50_ performance among all compared methods.

**Figure 7:**
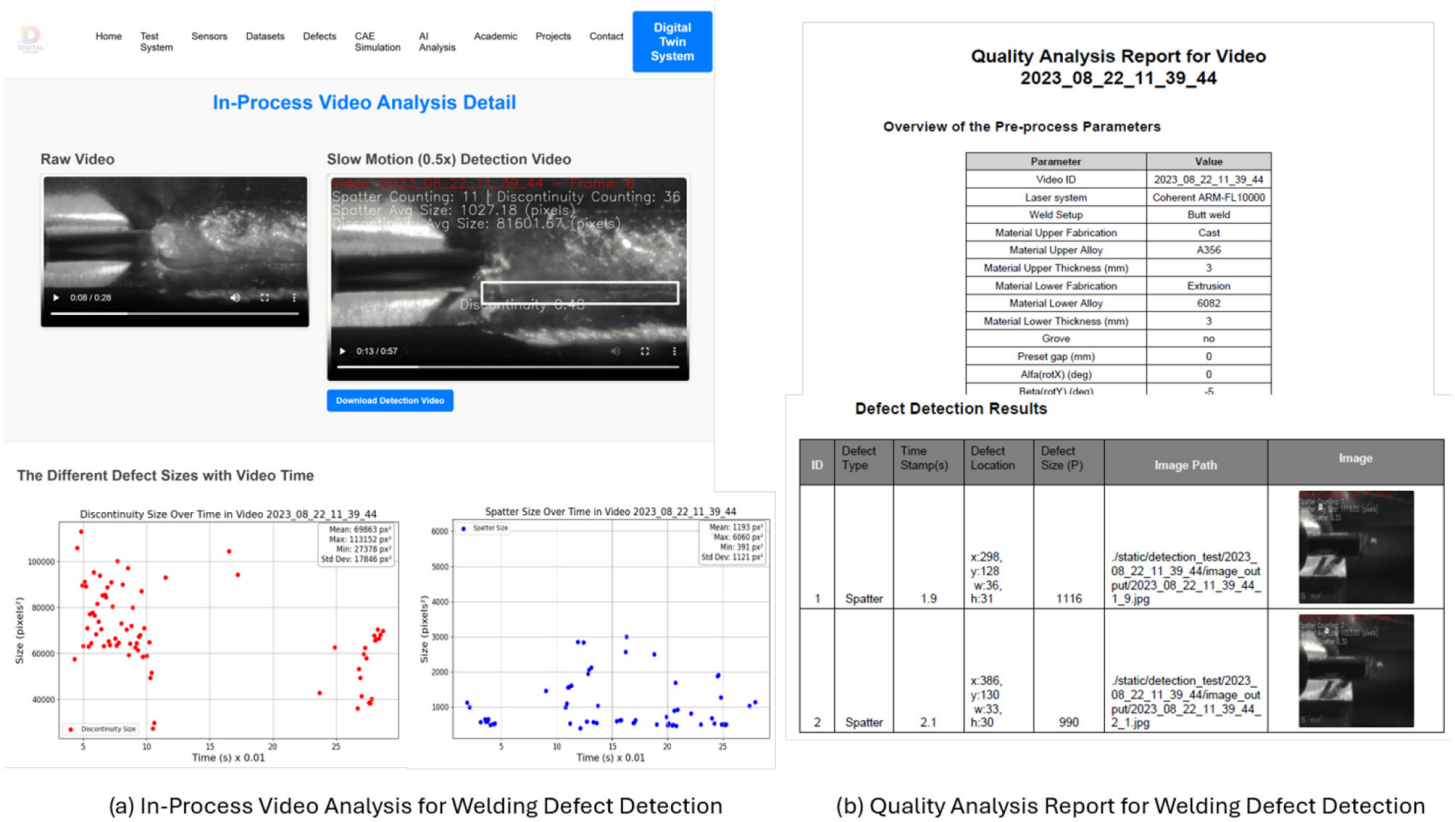
**(a)** In-process video analysis interface for welding defect detection and PAI modular. This interface displays raw and slow-motion annotated videos, alongside visual plots of defect size trends (e.g., discontinuities and spatter) over time, enabling quantitative analysis and traceability of defect progression during the welding process. **(b)** The report includes a comprehensive summary of pre-process parameters, spatial-temporal defect metadata (type, location, timestamp, size), and annotated defect images to support automated quality assessment and corrective action planning.

The processed results are shown on the video processing status dashboard (Figure 7). It includes a raw welding video and a slow-motion detection video that highlights detected defects such as spatter and discontinuities, along with real-time defect statistics. The size distribution of defects over time is visualized through discontinuity and spatter size plots, allowing for a quantitative assessment of welding quality. Users can download processed detection videos and defect size data for further inspection and analysis, and the defect analysis reports in Figure 7b, the report includes an overview of pre-process parameters, detailing material properties, welding setup, and laser system configurations, etc. The defect detection results section presents automatically identified defects, including their timestamps, spatial locations, and sizes, alongside corresponding annotated images. This structured analysis supports process optimization and quality assurance by enabling precise defect tracking and corrective actions. The interface enables users to access detailed defect reports, visualize defect trends, and refine detection accuracy. The system currently requires about one minute to analyse a 15-second video, making it more suitable for post-process inspection. This subsection explores the application of AI-driven defect detection and analysis in manufacturing. By integrating edge computing devices within practical manufacturing environments, where video data may be partially occluded and sensor signals can become noisy or misaligned, the system could incorporate multi-sensor data fusion to enhance robustness. Future developments will focus on improving reliability through training on noise-rich datasets and integrating physics-informed, context-aware adaptation mechanisms, thereby enabling early defect warnings through the combination of historical trends and live data stream

## 4 Conclusion and next steps

This paper presents a modular and scalable AI-enabled Digital Twin (AI-DT) framework that integrates Generative AI (GAI) and Predictive AI (PAI) to enhance real-time monitoring and defect detection in manufacturing. Although demonstrated within the context of welding, which includes sensor data acquisition, multi-model AI layers, and user interface components. The proposed framework can be adapted to other manufacturing scenarios, such as machining, assembly, and additive manufacturing, by reconfiguring sensor inputs, retraining the AI modules with domain-specific data, and updating process parameters to reflect task-specific operational conditions.

As manufacturing environments are inherently noisy and unpredictable, several limitations remain. Future developments will focus on enhancing the robustness and adaptability of our AI models, incorporate metamodeling approaches and automation workflow (Stavropoulos et al., 2023). Training on diverse datasets that capture noise, occlusion, and variability will strengthen model resilience, while metamodels and workflow databases can accelerate Digital Twin development by enabling modular reuse, systematic workflow design, and alignment with business and policy constraints. Furthermore, we plan to integrate Explainable AI (XAI) for actionable root cause analysis, deploy Context-Aware AI to support real-time parameter optimization, and incorporate Agentic AI to facilitate autonomous decision-making and adaptive control. Collectively, these enhancements will advance the scalability, reliability, and practical applicability of the proposed framework across a wide range of industrial scenarios.

## Data Availability

The datasets presented in this article are not readily available because as those data are used for the BMW, we should apply from BMW. Requests to access the datasets should be directed to d.dai@aston.ac.uk.
