# Supplementary figures and images for: Generative and Predictive AI for digital twin systems in manufacturing
Source: Front Artif Intell. 2025 Dec 17;8:1655470. doi: 10.3389/frai.2025.1655470 (PMC12753877; doi:10.3389/frai.2025.1655470)

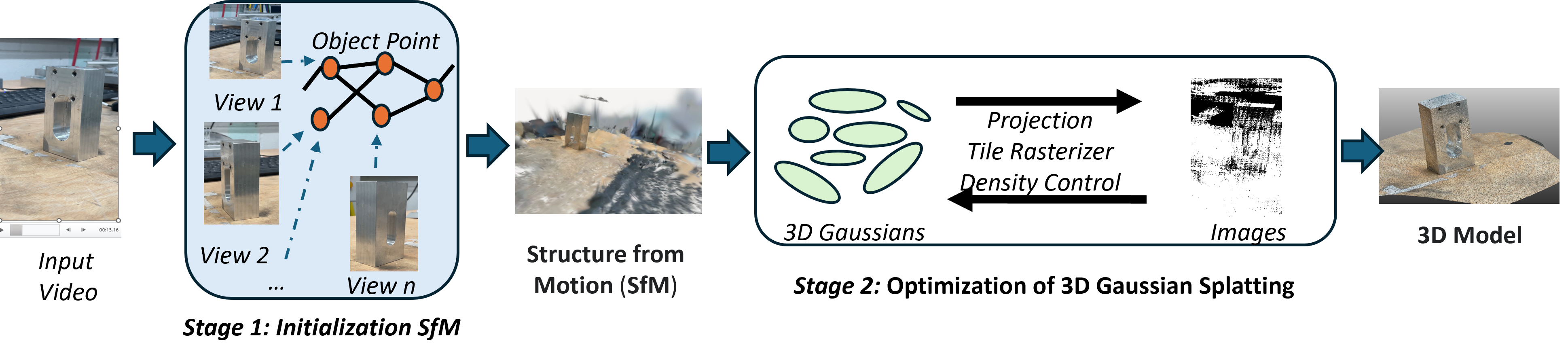

Supplement: Supplementary file 1 [file Presentation_1.zip › figure_folder/3DGS.png]

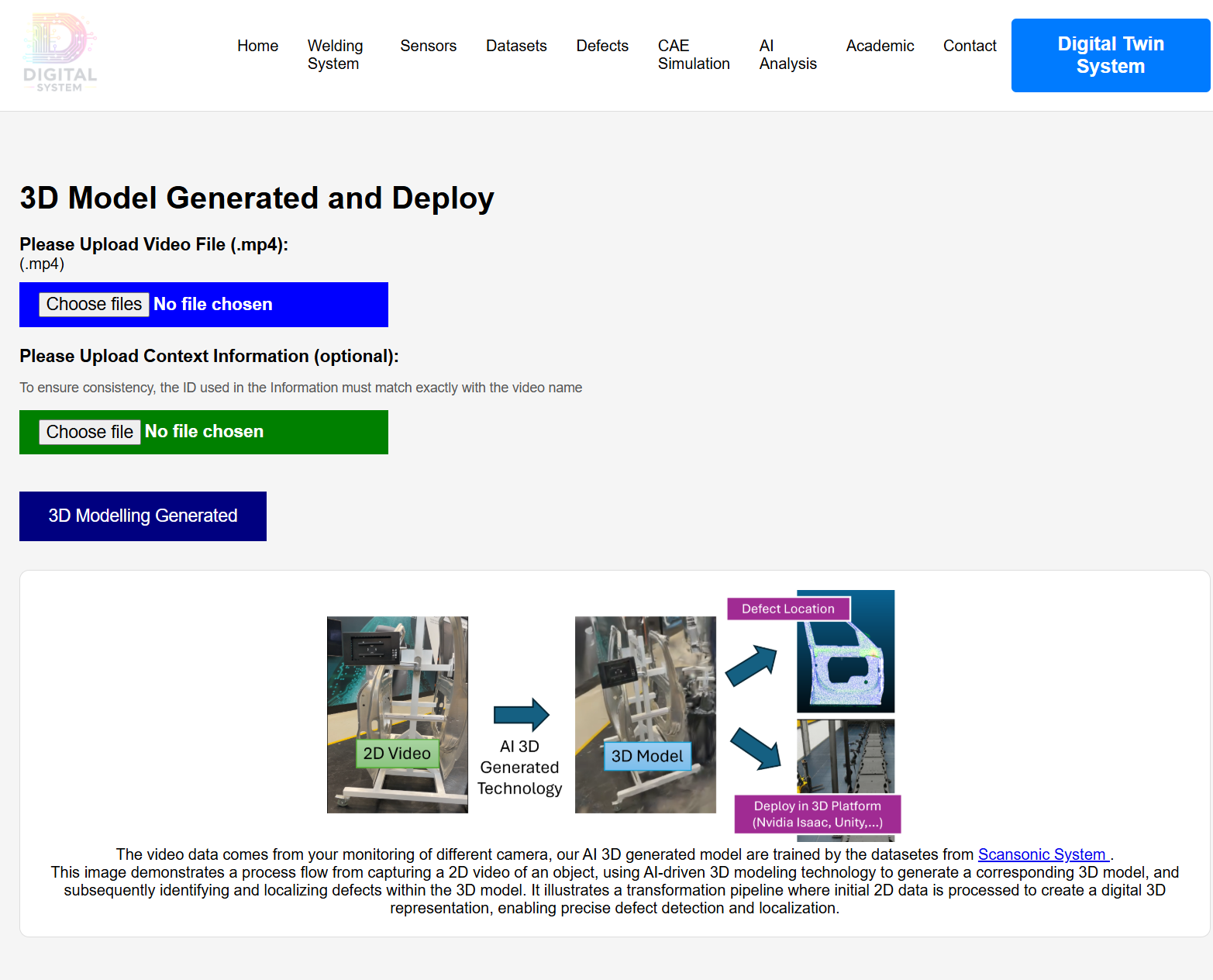

Supplement: Supplementary file 1 [file Presentation_1.zip › figure_folder/3D_Modeling.png]

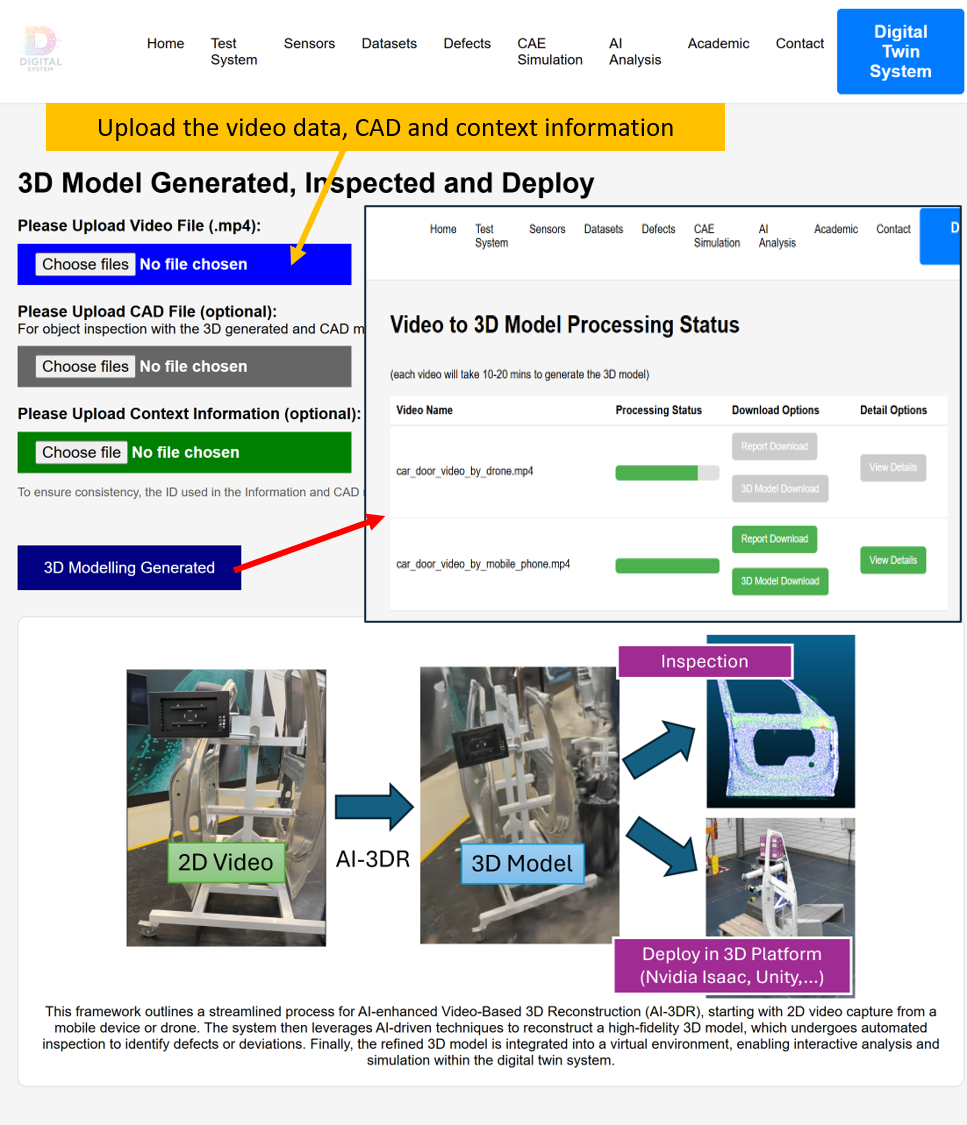

Supplement: Supplementary file 1 [file Presentation_1.zip › figure_folder/3D_Modeling_results.png]

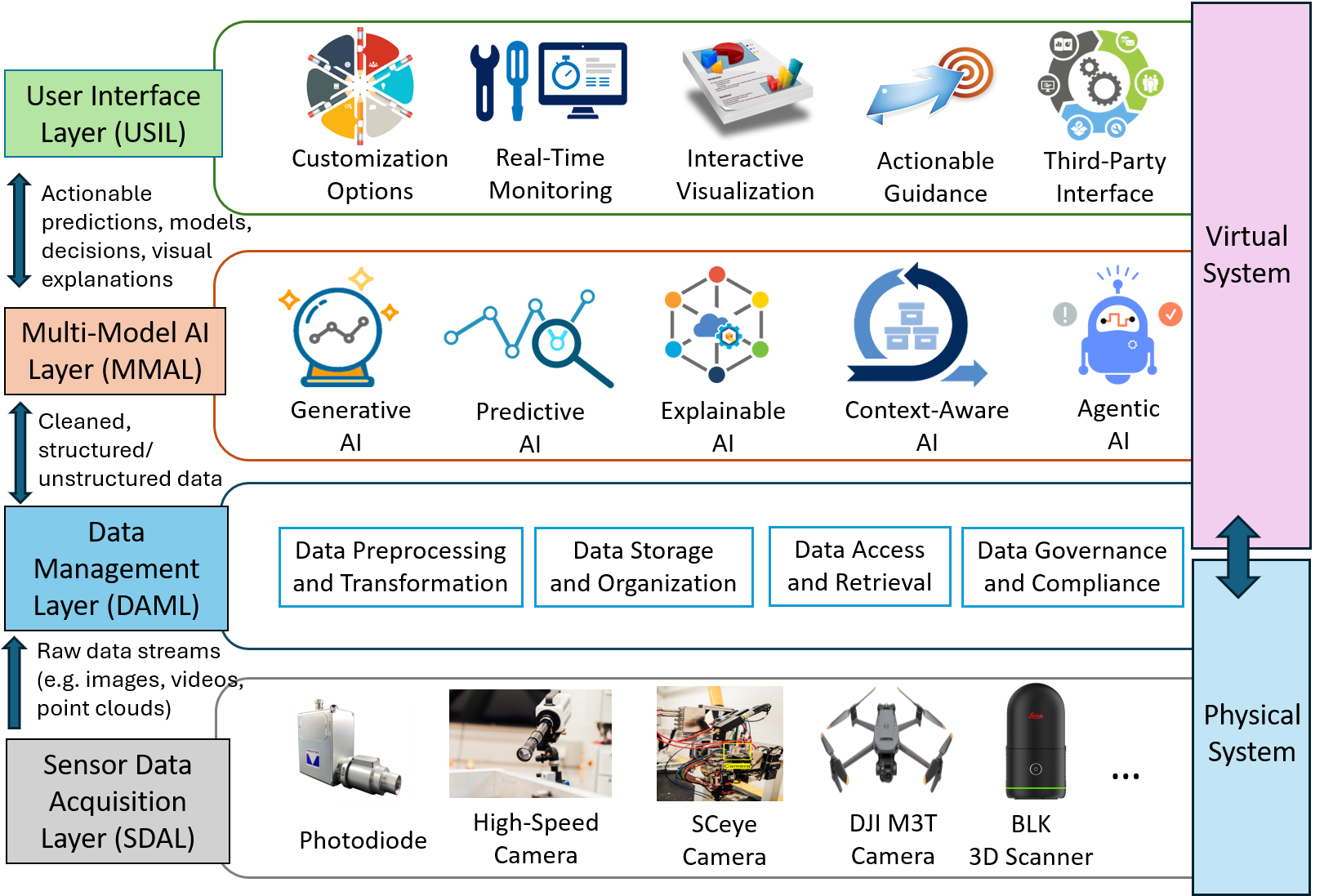

Supplement: Supplementary file 1 [file Presentation_1.zip › figure_folder/AI-DT framework.png]

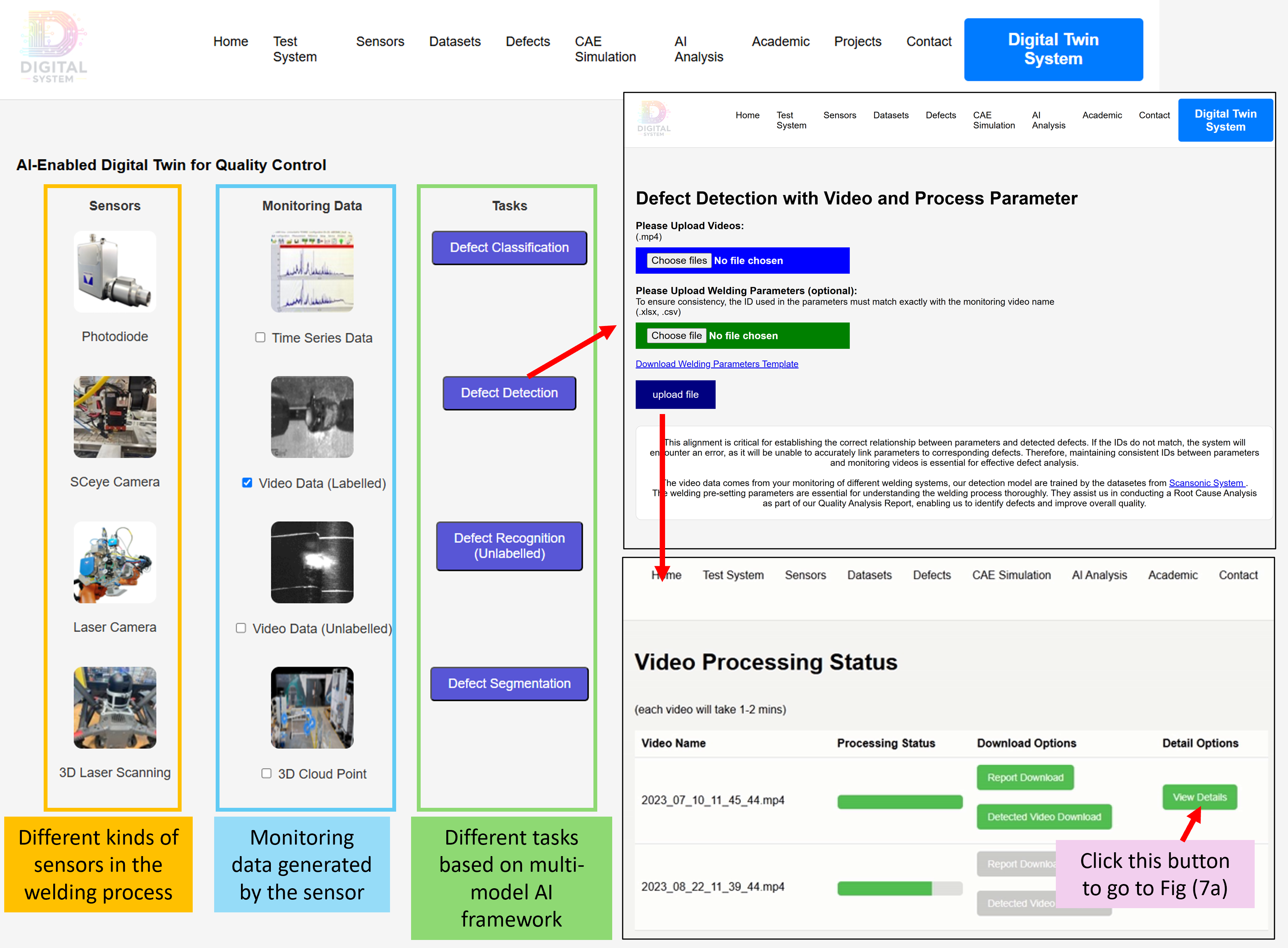

Supplement: Supplementary file 1 [file Presentation_1.zip › figure_folder/AI_analysis_results.png]

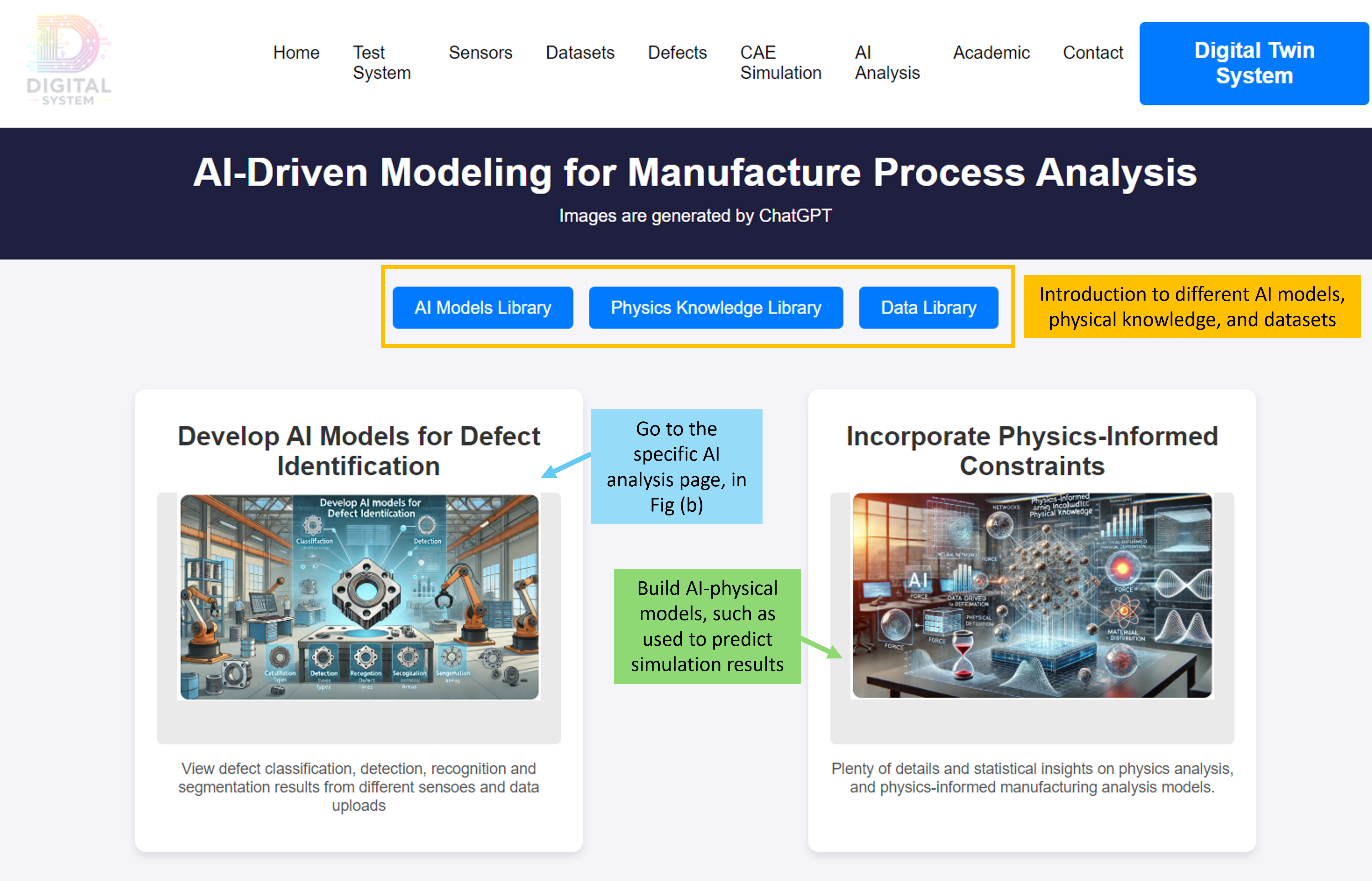

Supplement: Supplementary file 1 [file Presentation_1.zip › figure_folder/AI_Driven_Modeling.png]

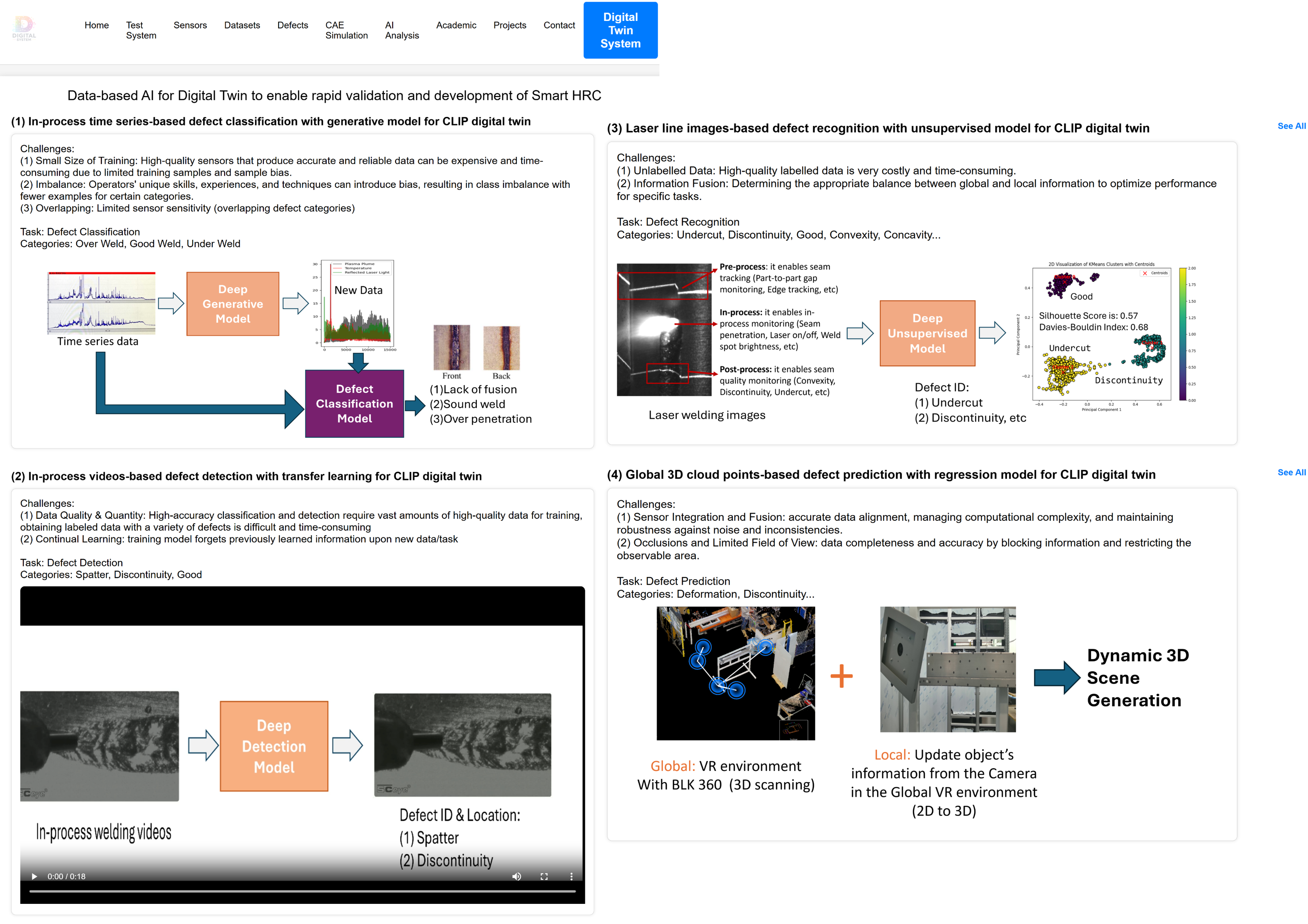

Supplement: Supplementary file 1 [file Presentation_1.zip › figure_folder/AI_summary.png]

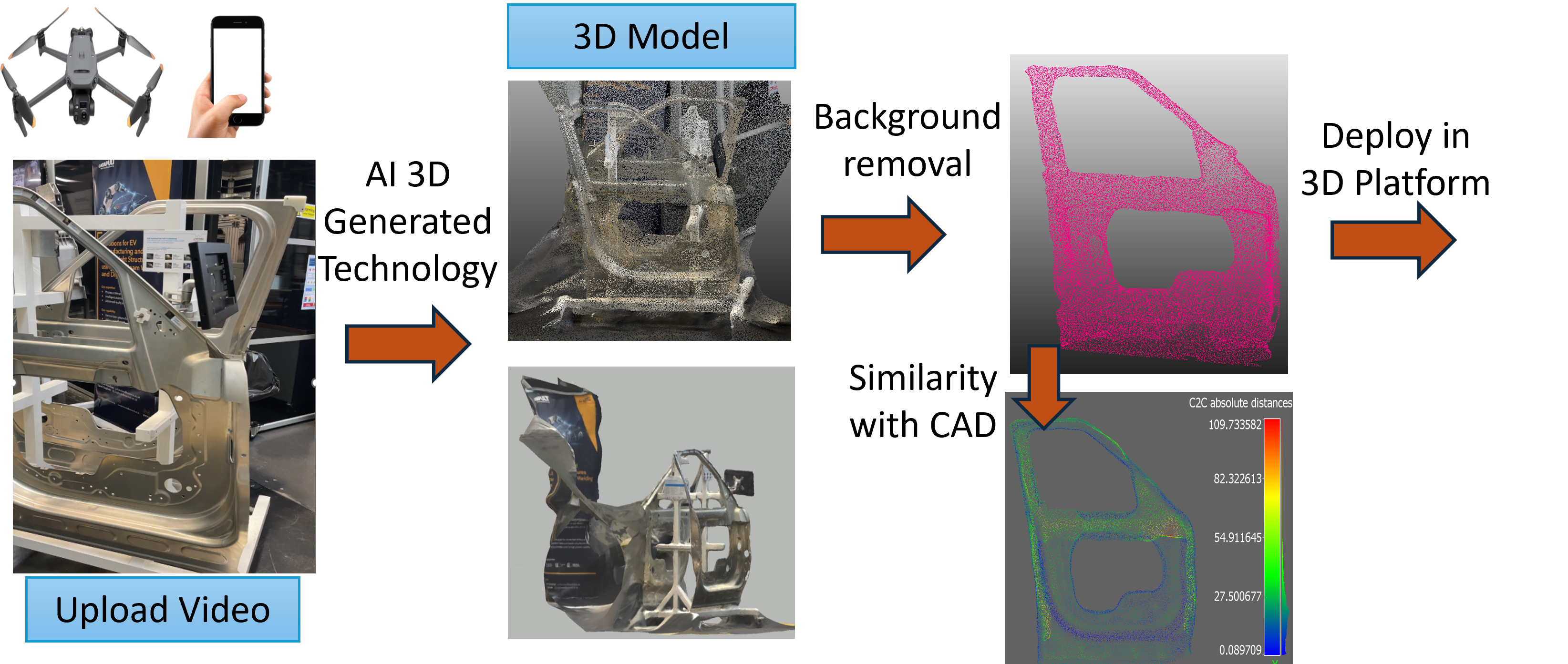

Supplement: Supplementary file 1 [file Presentation_1.zip › figure_folder/car_door_result.png]

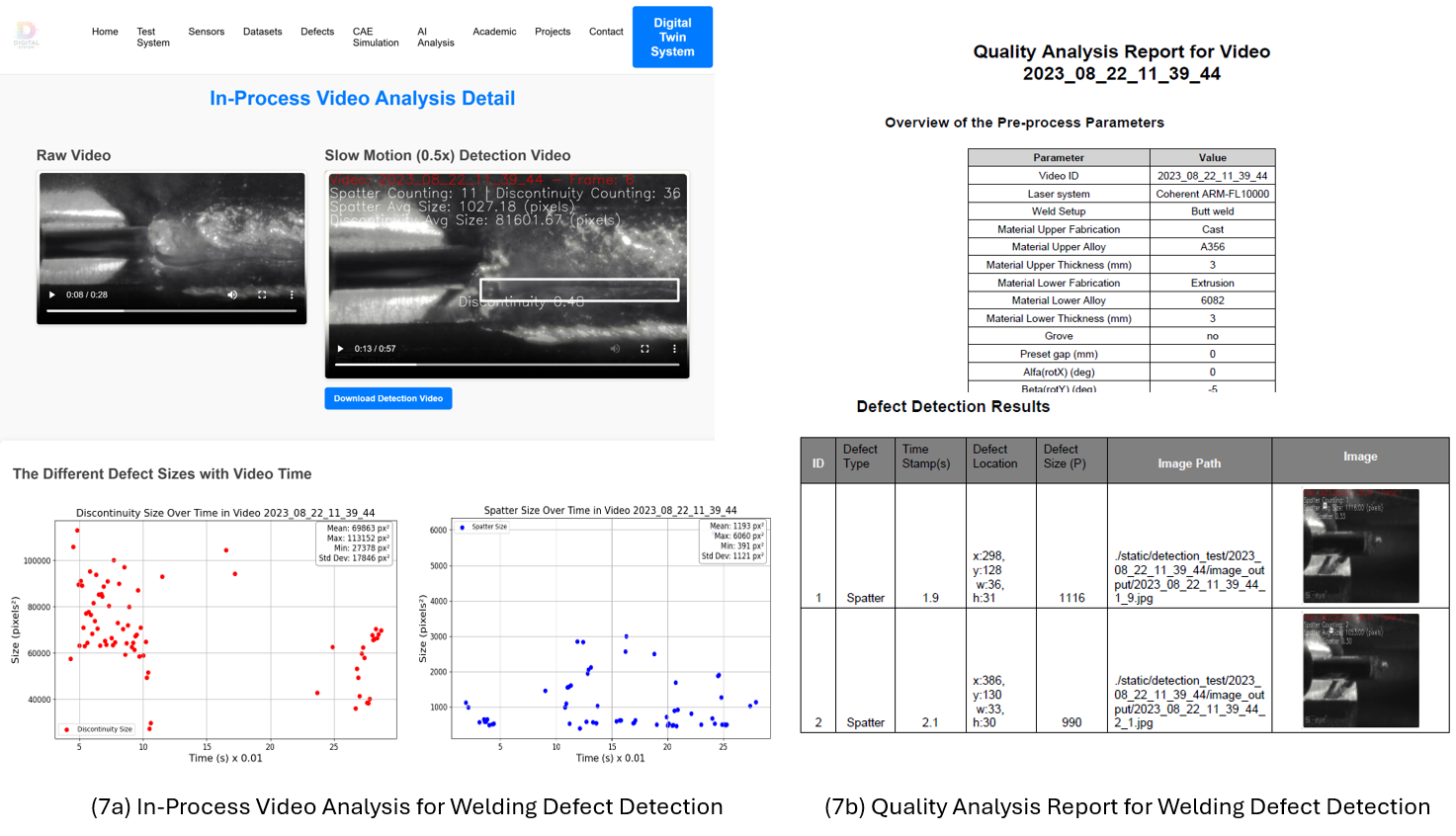

Supplement: Supplementary file 1 [file Presentation_1.zip › figure_folder/detail_show_videoName=2023_08_22_11_39_44.png]

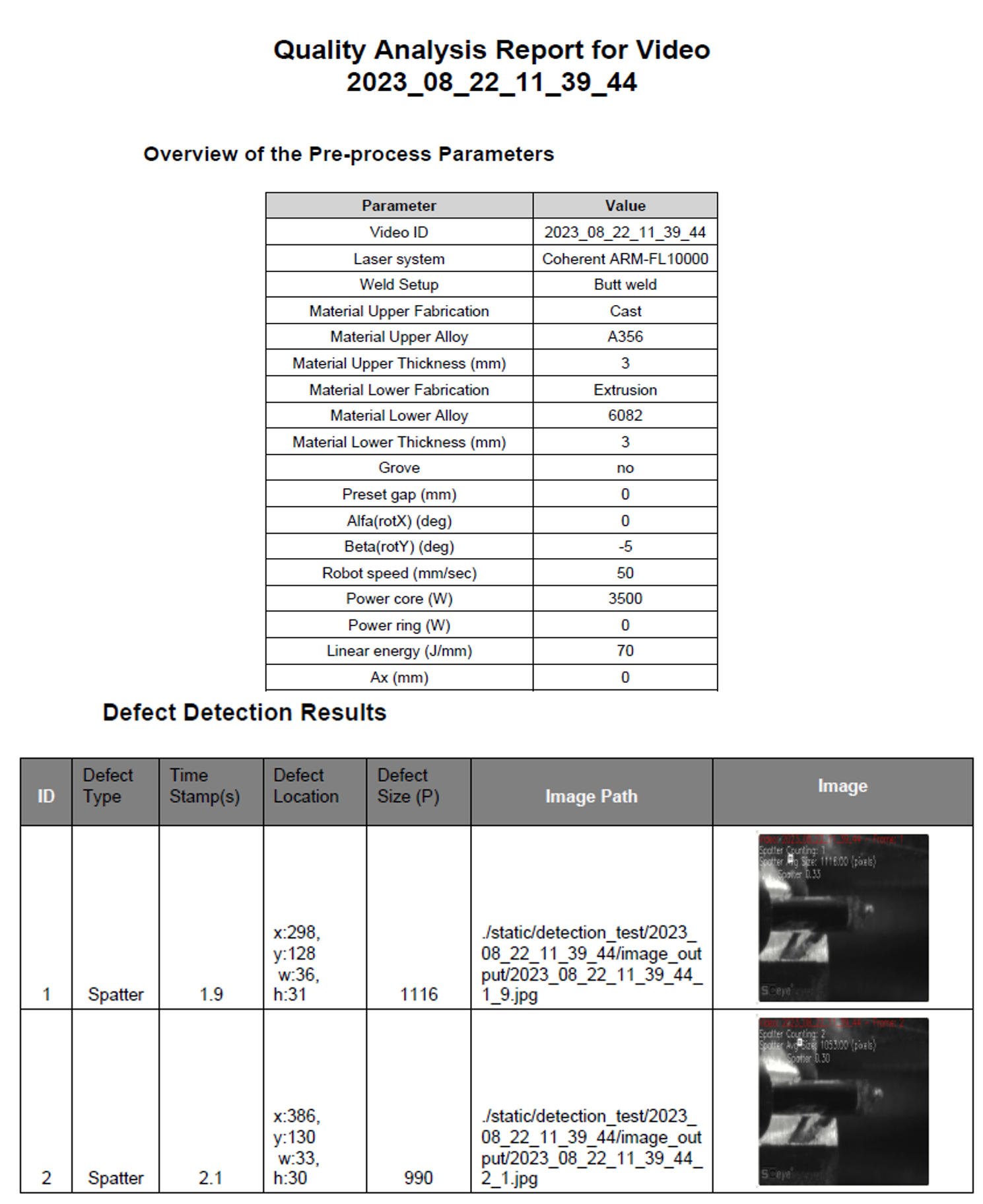

Supplement: Supplementary file 1 [file Presentation_1.zip › figure_folder/detect-report.png]

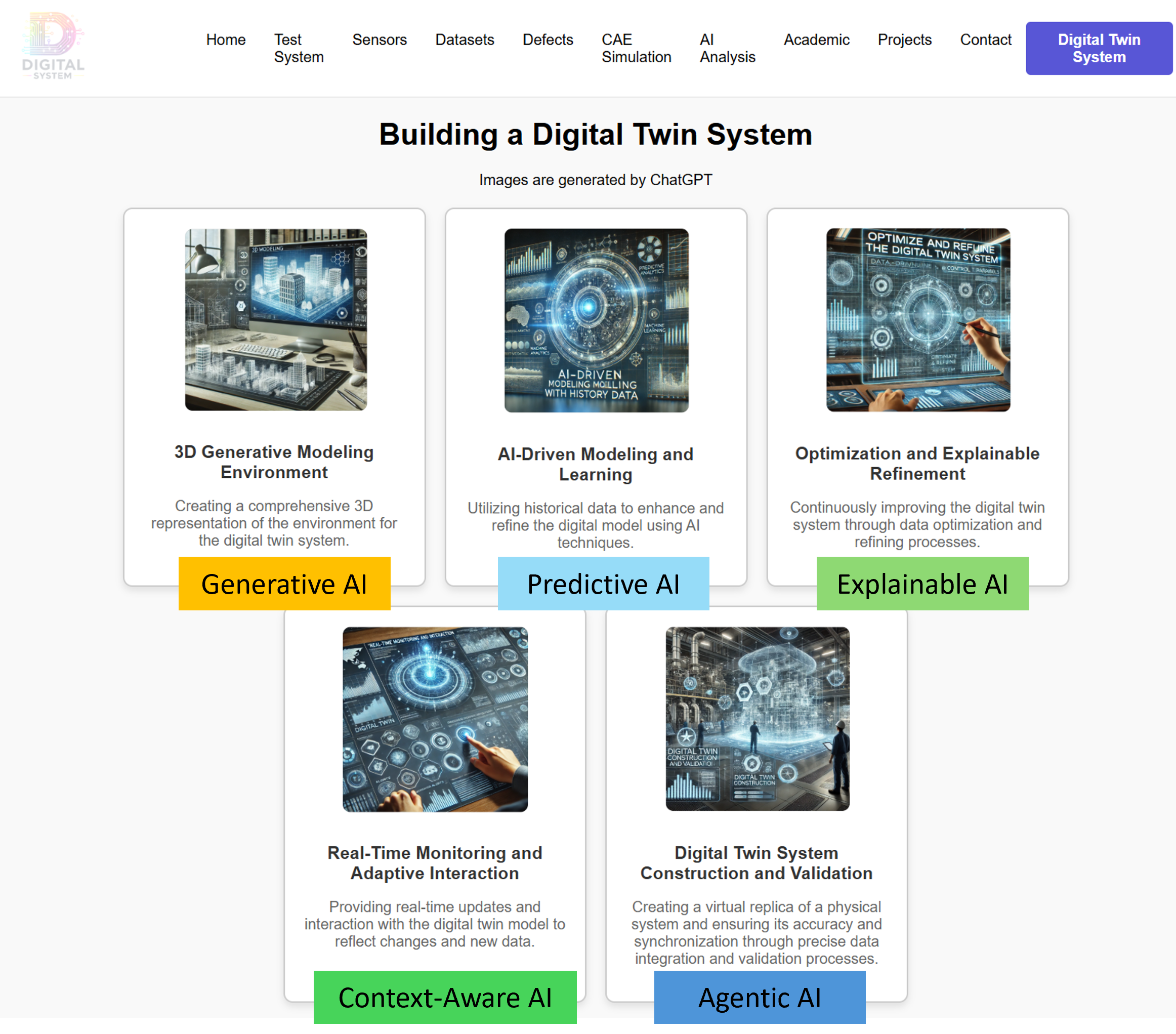

Supplement: Supplementary file 1 [file Presentation_1.zip › figure_folder/DT_main.png]

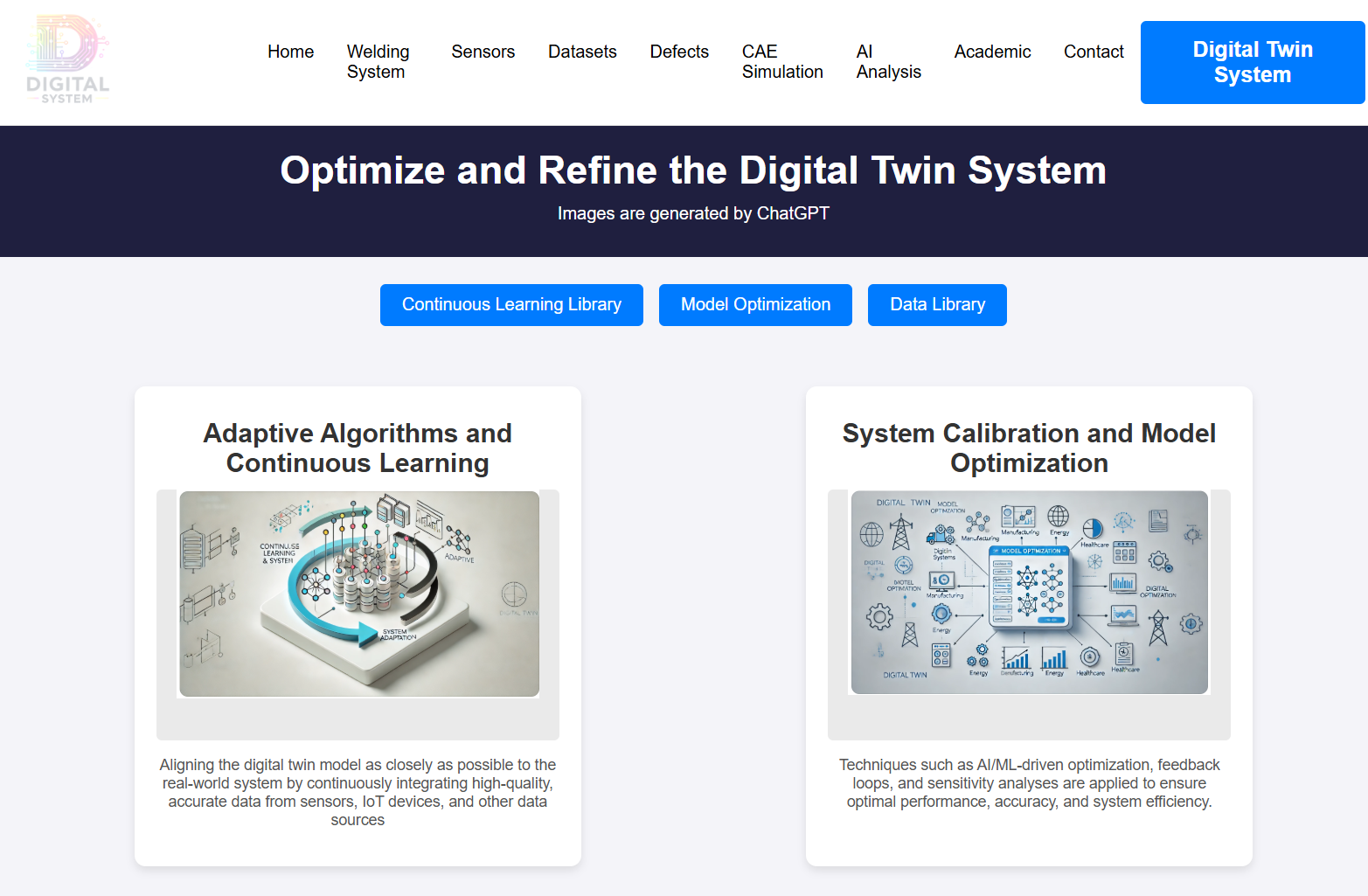

Supplement: Supplementary file 1 [file Presentation_1.zip › figure_folder/Optimize_Refine_System.png]

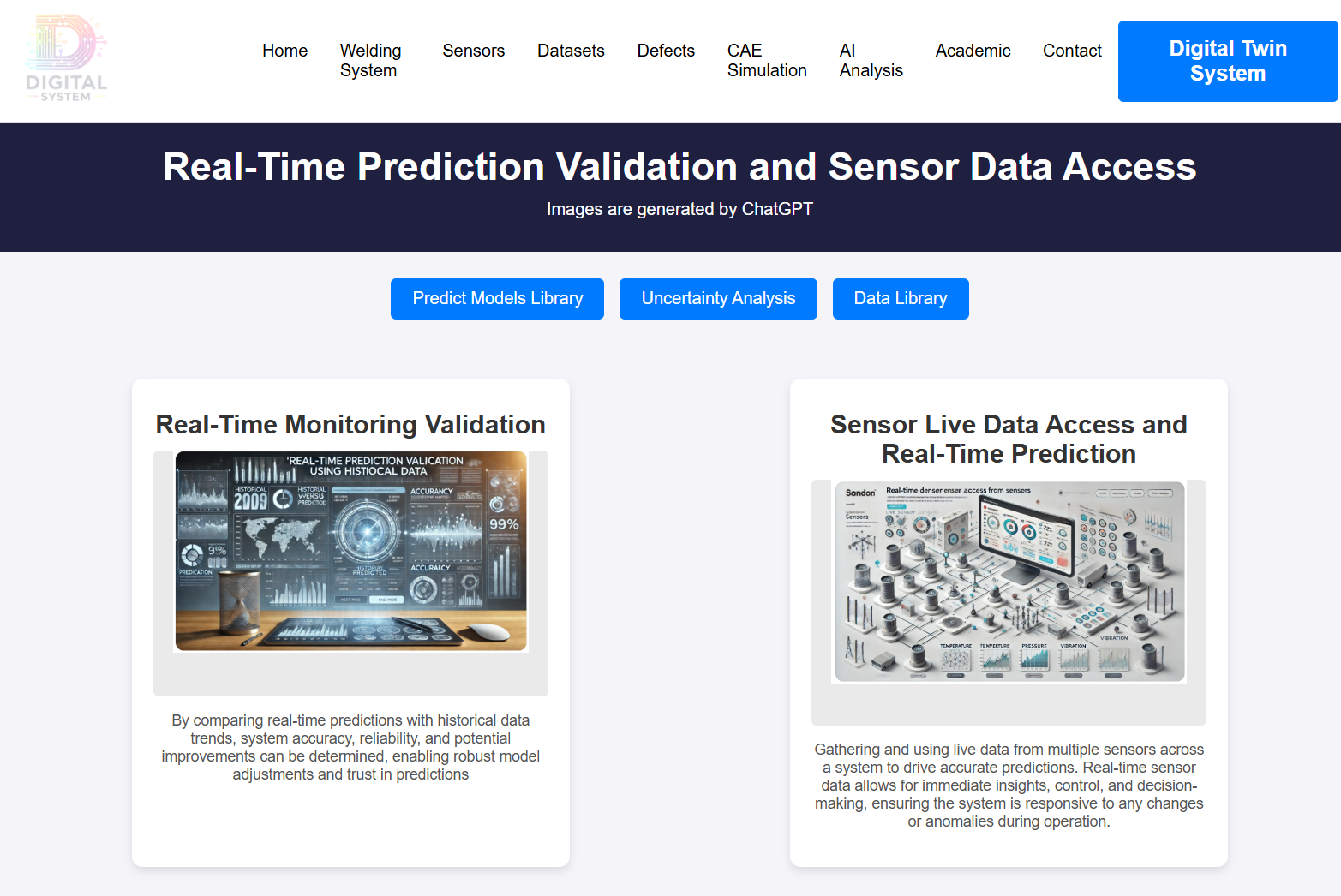

Supplement: Supplementary file 1 [file Presentation_1.zip › figure_folder/Real_Time_Prediction.png]

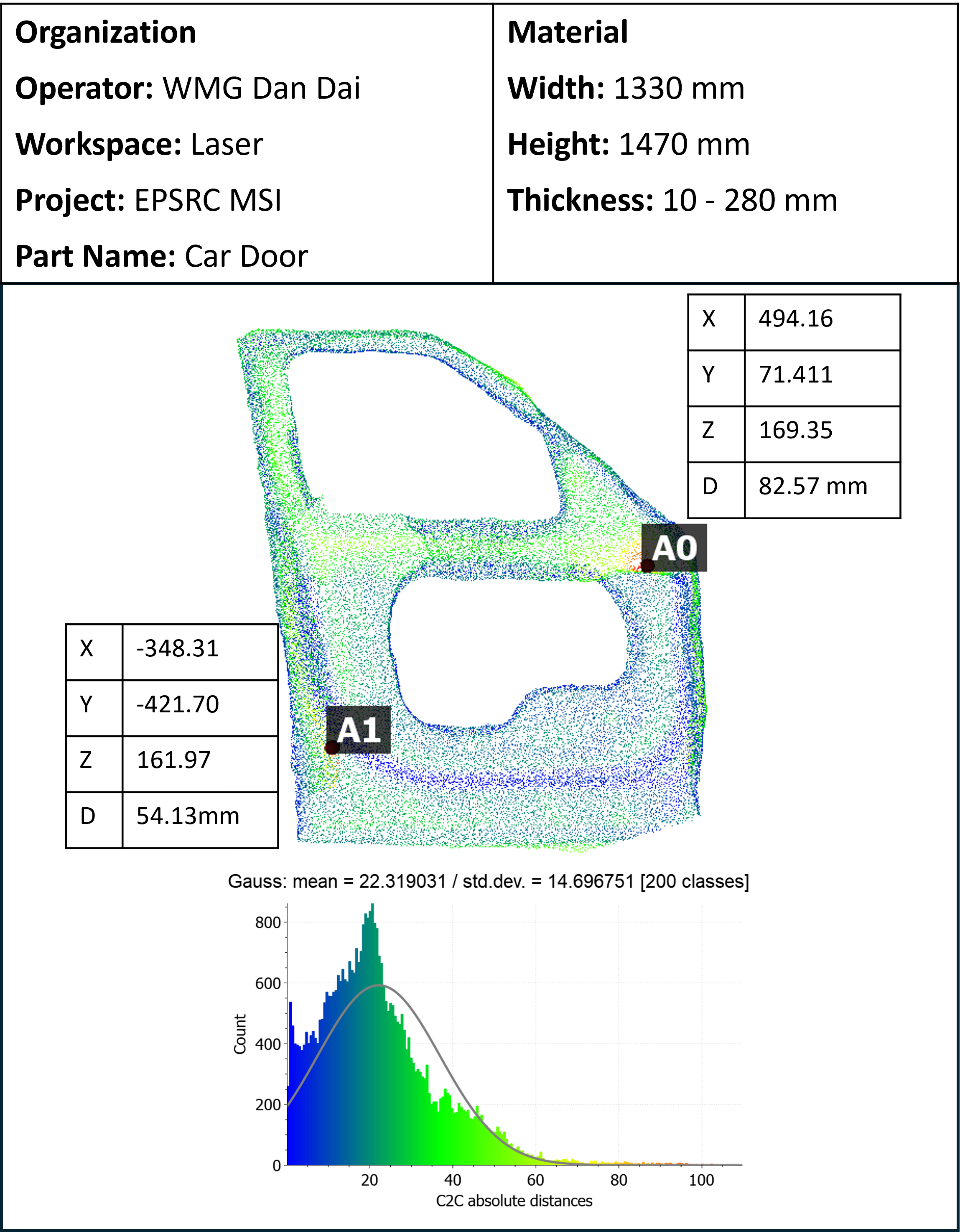

Supplement: Supplementary file 1 [file Presentation_1.zip › figure_folder/Report_3D.png]

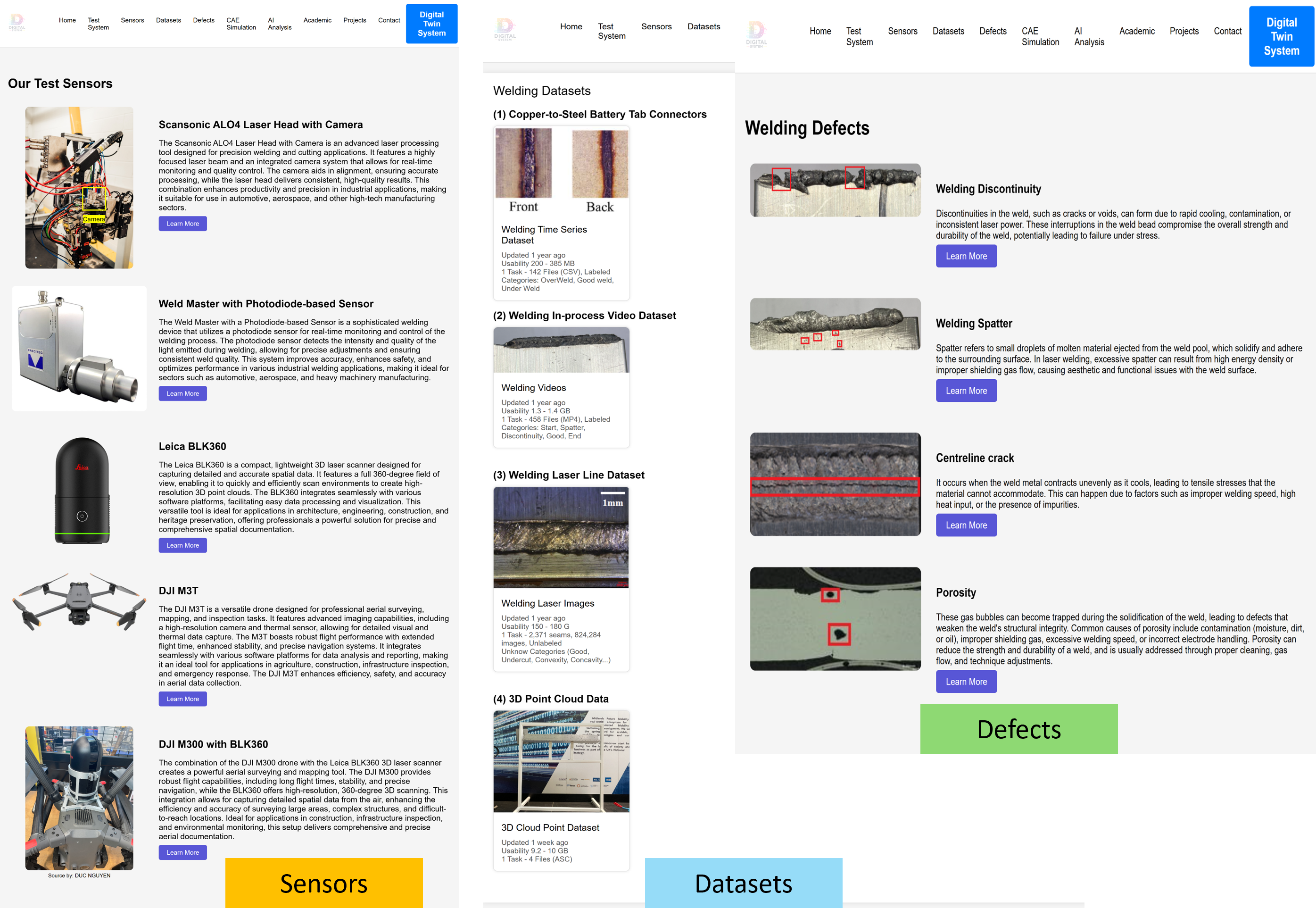

Supplement: Supplementary file 1 [file Presentation_1.zip › figure_folder/sensor_data_defect.png]

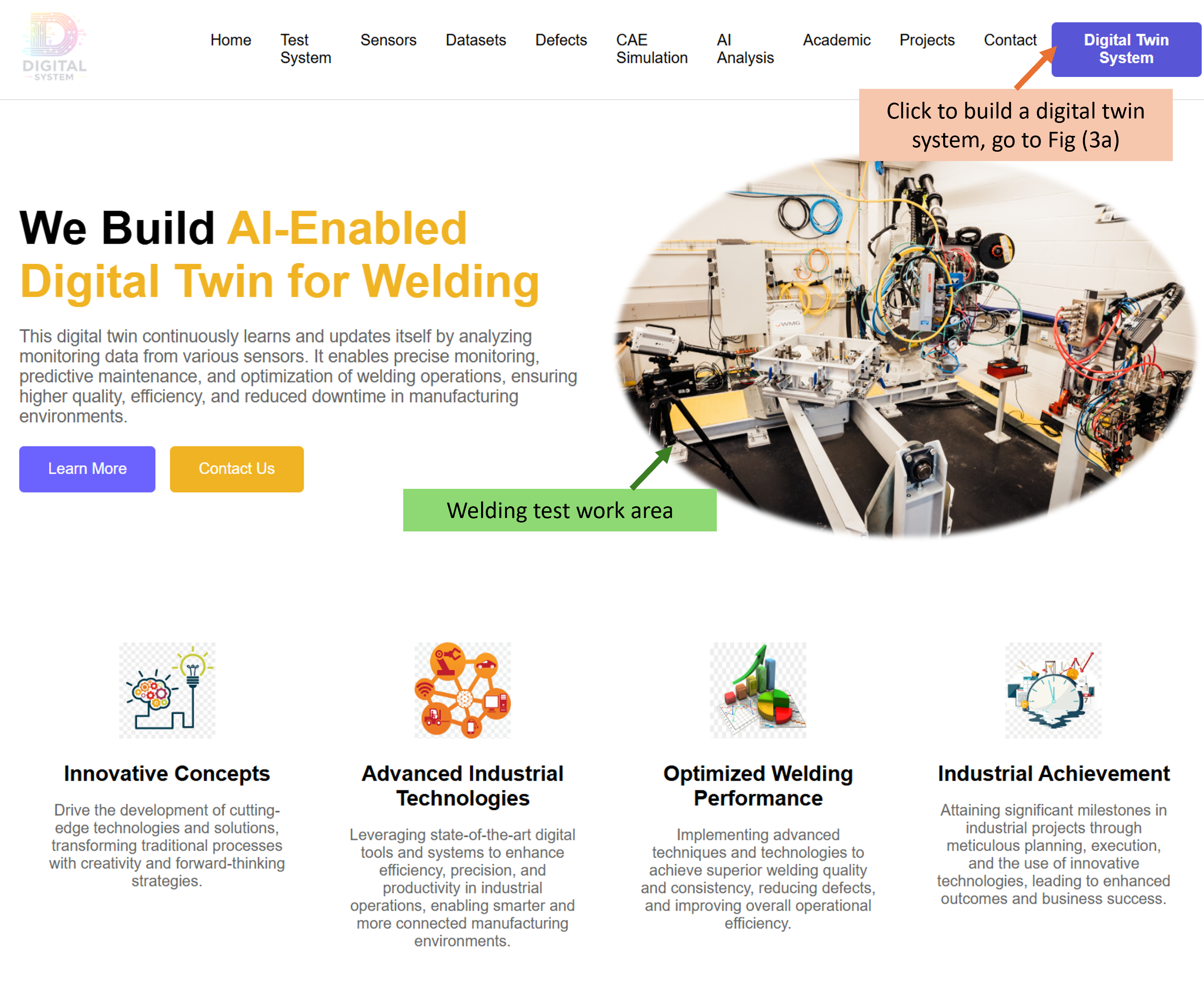

Supplement: Supplementary file 1 [file Presentation_1.zip › figure_folder/sys_main.png]

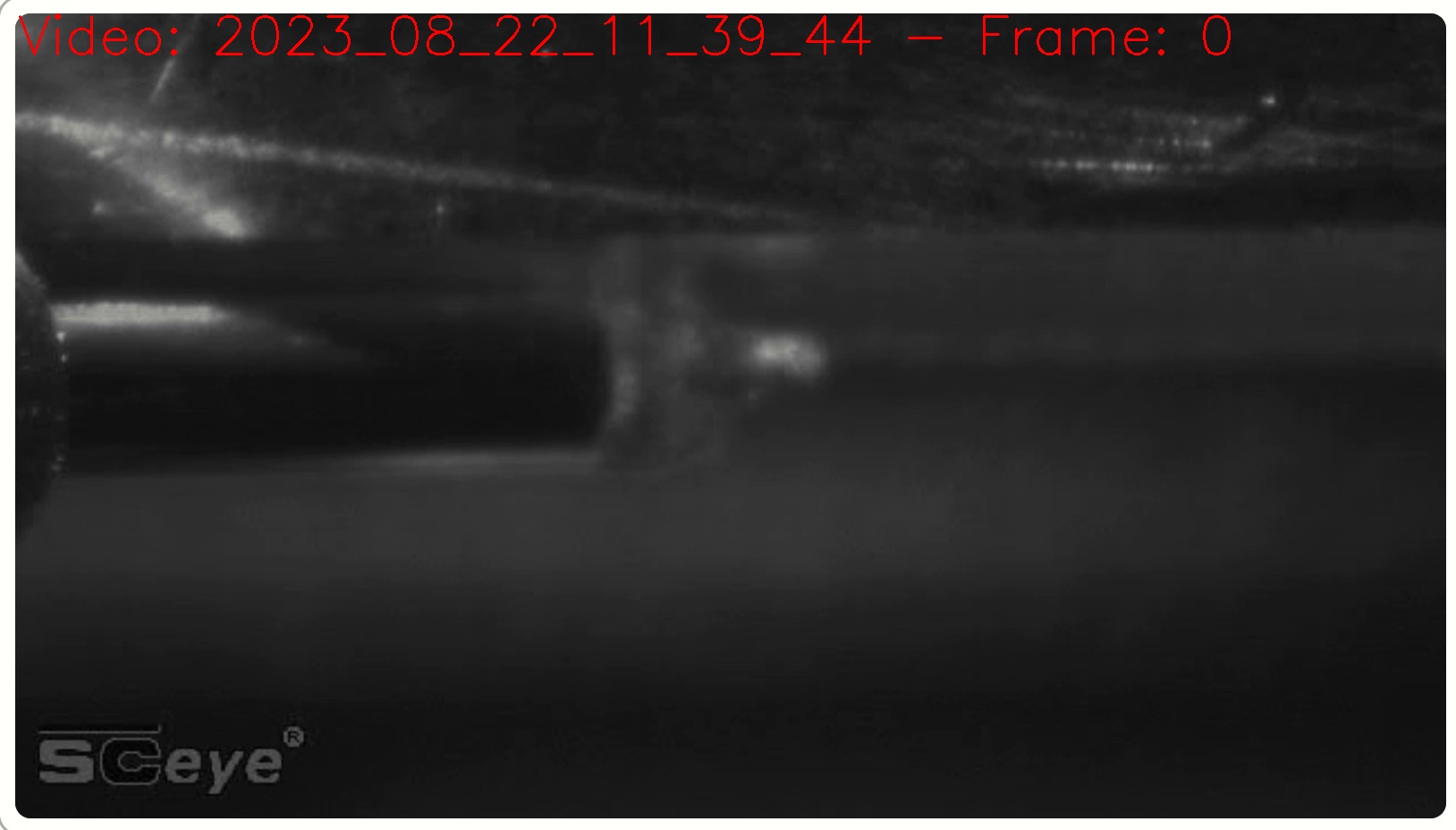

Supplement: Supplementary file 1 [file Presentation_1.zip › figure_folder/video_detected/2023_08_22_11_39_44_0_1.jpg]

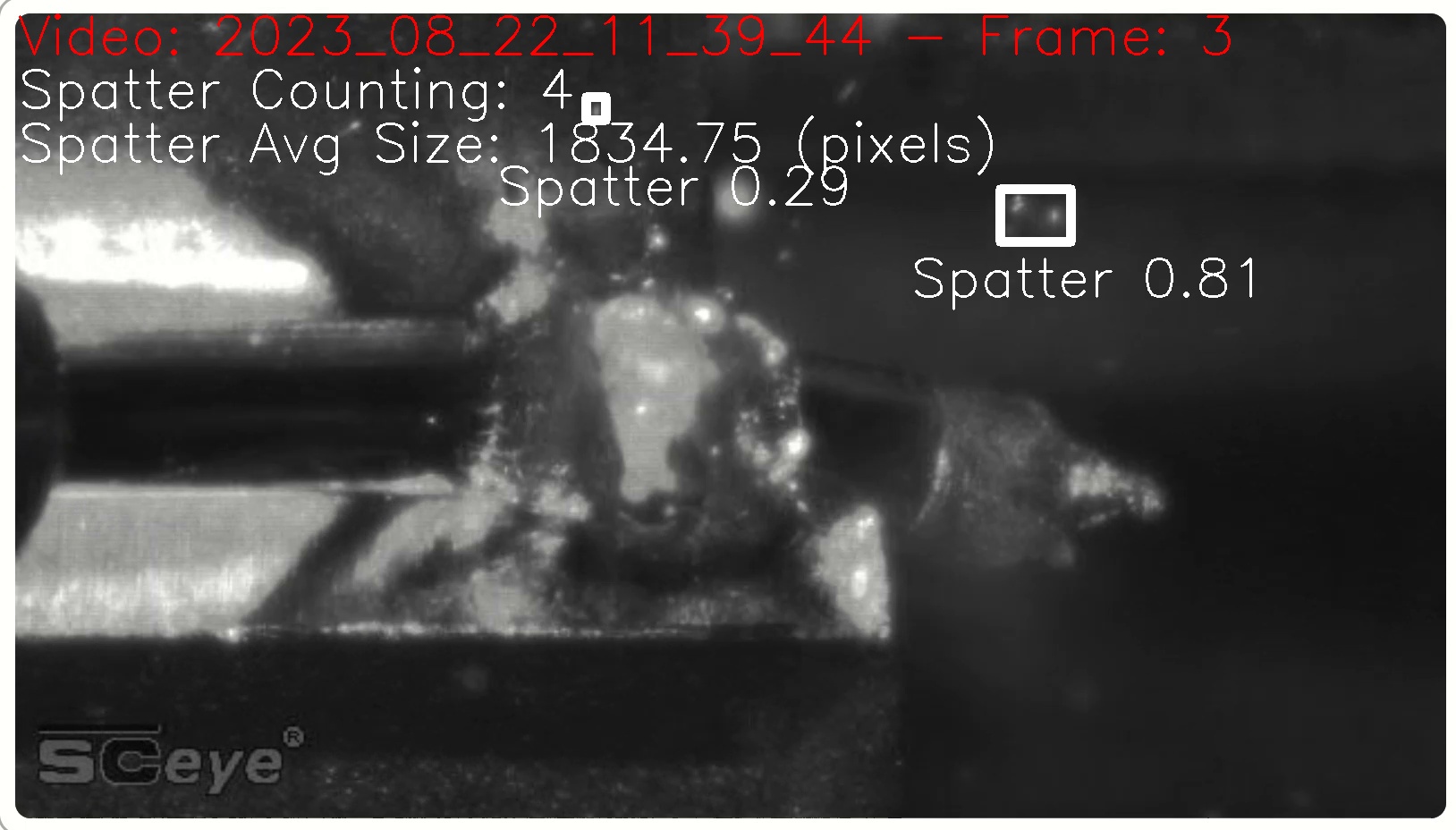

Supplement: Supplementary file 1 [file Presentation_1.zip › figure_folder/video_detected/2023_08_22_11_39_44_3_1.jpg]

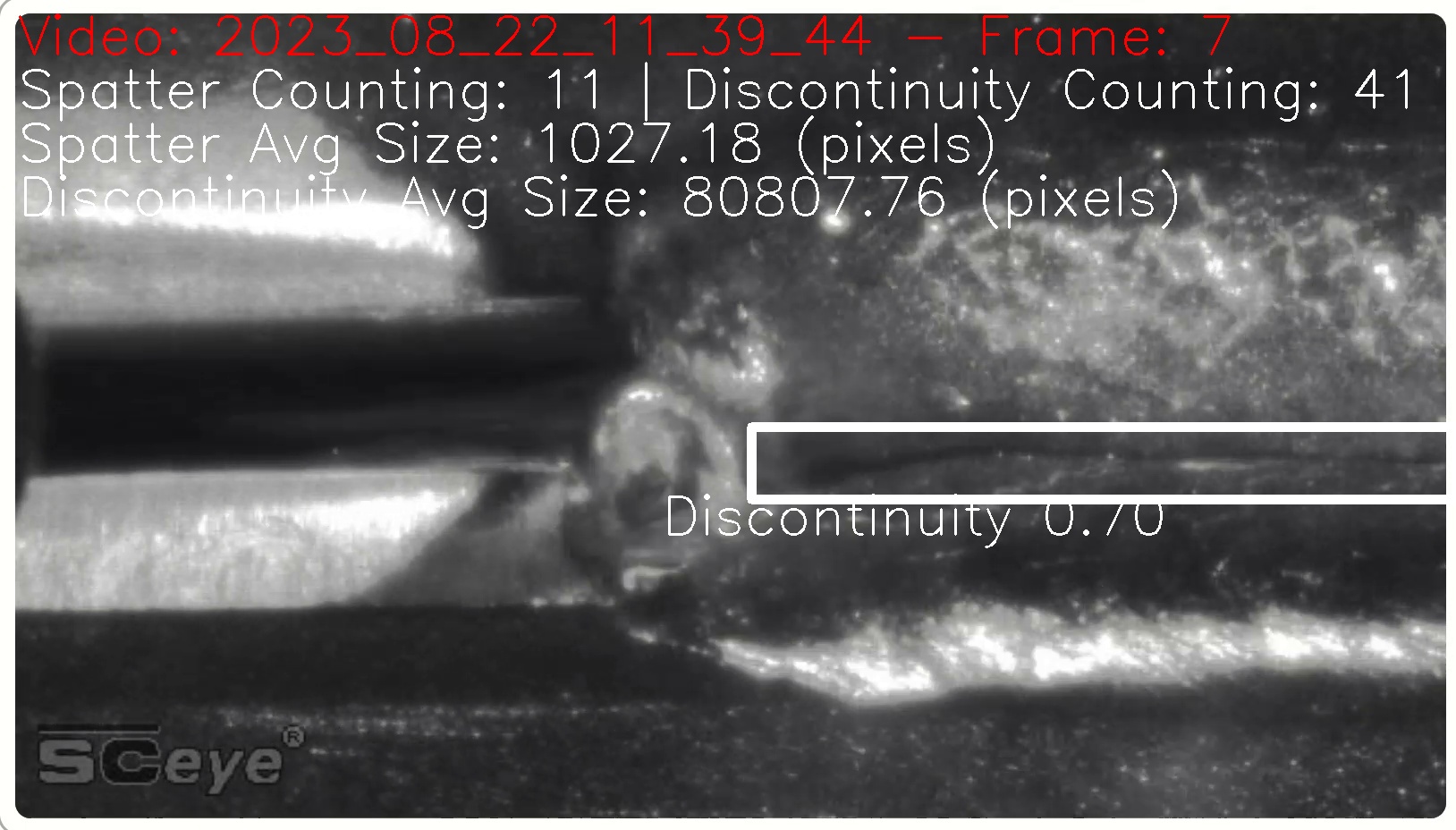

Supplement: Supplementary file 1 [file Presentation_1.zip › figure_folder/video_detected/2023_08_22_11_39_44_7_1.jpg]

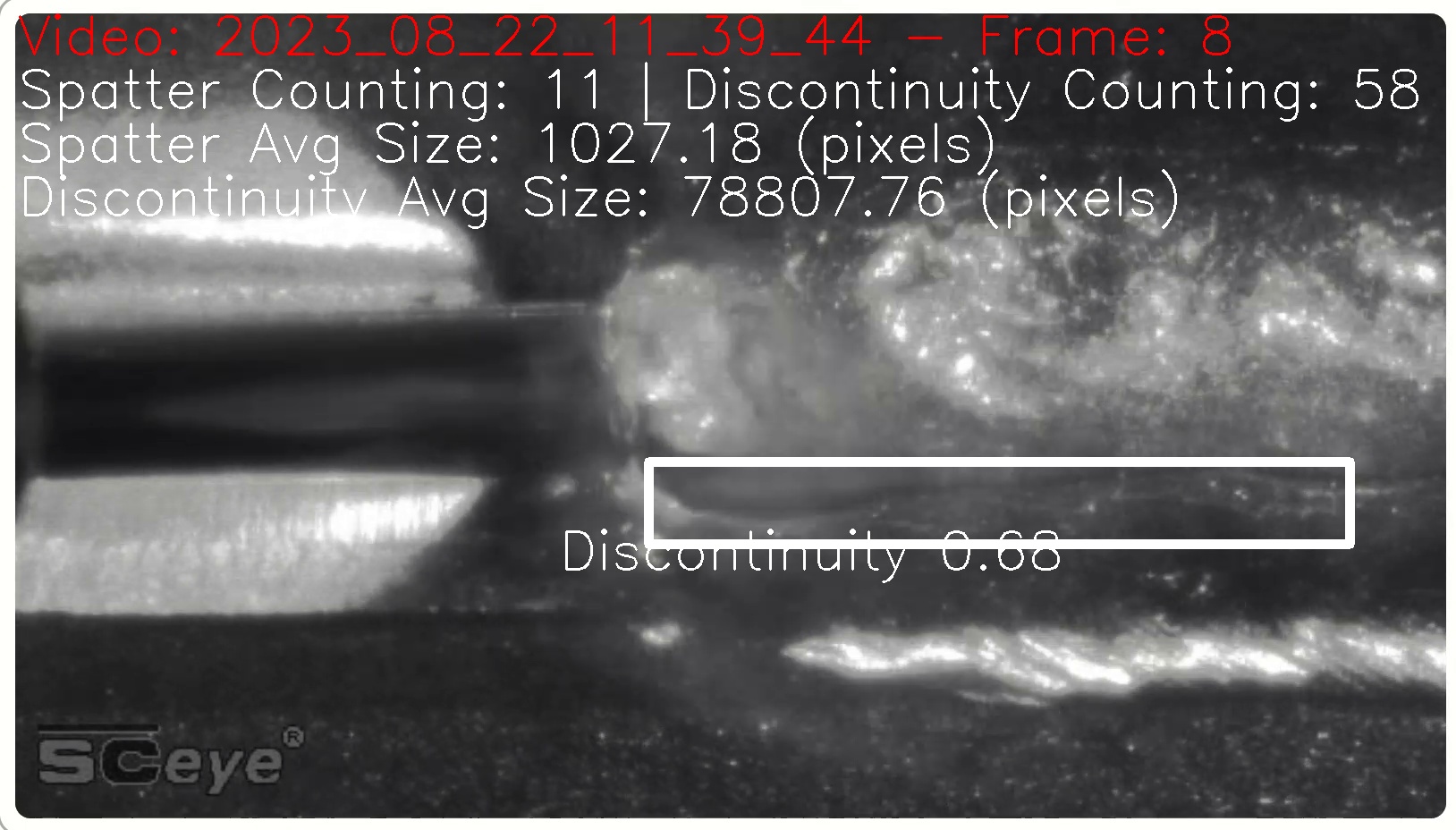

Supplement: Supplementary file 1 [file Presentation_1.zip › figure_folder/video_detected/2023_08_22_11_39_44_8_8.jpg]

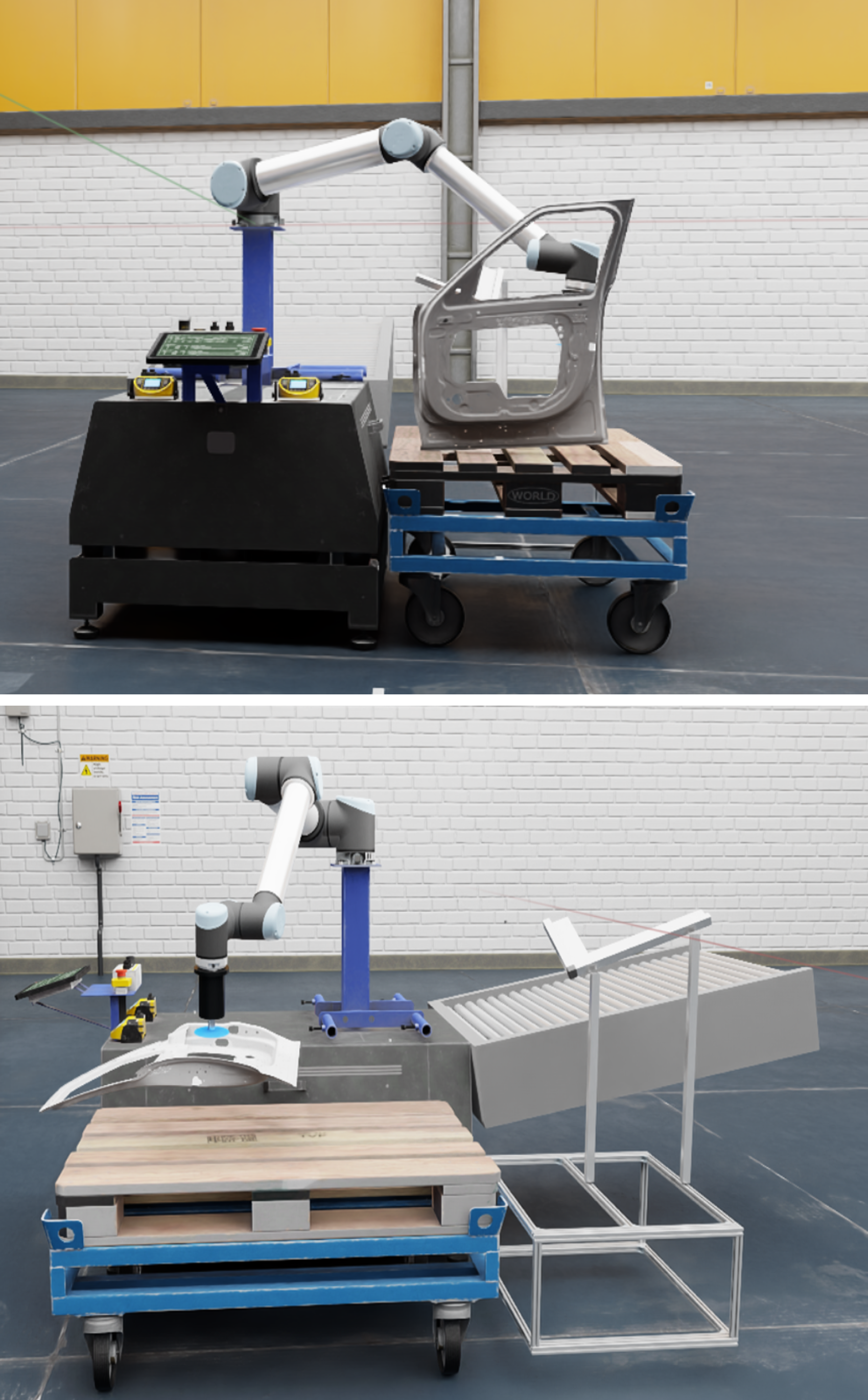

Supplement: Supplementary file 1 [file Presentation_1.zip › figure_folder/virtual envir.png]

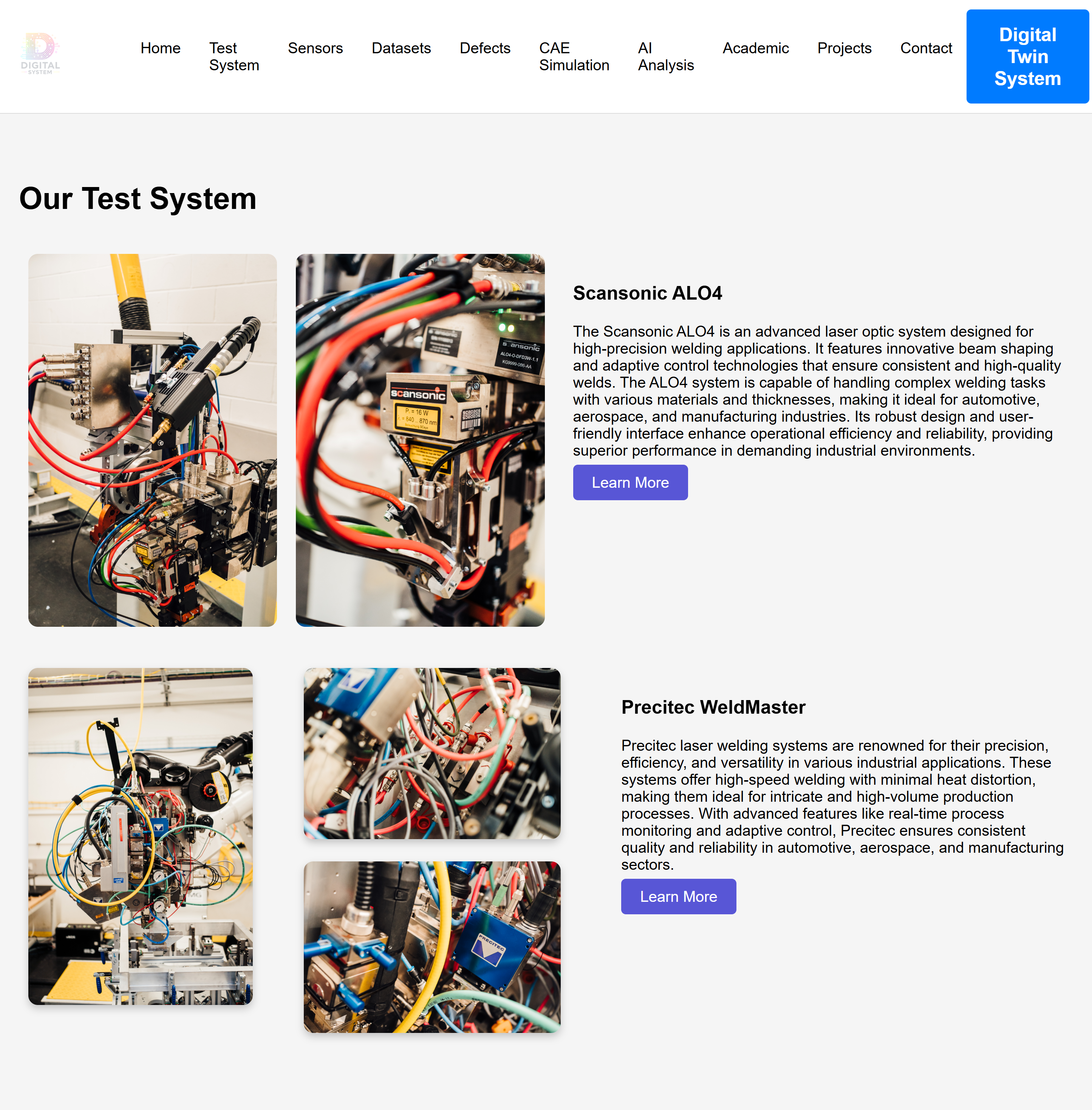

Supplement: Supplementary file 1 [file Presentation_1.zip › figure_folder/welding_system.png]
